# Bacterial Biohybrids With Dual Magnet and Hypoxia Tropism for Ferroptosis Activation in Deep Tumor Regions

**DOI:** 10.1002/EXP.20240287

**Published:** 2026-02-10

**Authors:** Sijie Shao, Huilan Zhuang, Tingjie Bai, Xuemei Zeng, Angelo Homayoun All, Shuangqian Yan

**Affiliations:** ^1^ Strait Institute of Flexible Electronics (SIFE Future Technologies) Fujian Key Laboratory of Flexible Electronics Fujian Normal University and Strait Laboratory of Flexible Electronics (SLoFE) Fuzhou China; ^2^ Key Laboratory of Microbial Pathogenesis and Interventions of Fujian Province University, Biomedical Research Center of South China College of Life Sciences Fujian Normal University Fuzhou P. R. China; ^3^ Department of Chemistry Hong Kong Baptist University Hong Kong SAR China

**Keywords:** bacterial biohybrids, ferroptosis, hypoxia, tumor penetration

## Abstract

Effectively delivering therapeutics to deep tumor regions is crucial for successful treatment but remains a formidable challenge. The severe hypoxia common in these areas can inhibit various therapeutic‐induced cell death pathways, including ferroptosis. Herein, we present the design of a bacterial biohybrid (Ec@ZFOY) with dual magnetic and hypoxic tropism for targeted therapeutic delivery and ferroptosis activation in the deep tumor region. This biohybrid is constructed using hypoxia‐targeted *Escherichia coli* and magnetic Zn_0.16_Fe_1.24_O_4_ (ZFO) nanoparticles loaded with YC‐1, a hypoxia‐inducible factor‐1α (HIF‐1α) inhibitor. ZFO exhibits peroxidase‐ and glutathione oxidase‐mimetic activities, catalyzing tumor‐derived H_2_O_2_ into hydroxyl radical and inhibiting glutathione peroxidase 4, thereby inducing ferroptosis. Additionally, Ec@ZFOY demonstrates pH‐responsive YC‐1 release, which inhibits HIF‐1α and reduces lipid droplets, enhancing ferroptosis through the release of polyunsaturated fatty acids. Both in vitro and in vivo experiments confirm the significant therapeutic efficacy of Ec@ZFOY. This study unveils the design of magnetically and hypoxia‐tropic bacterial biohybrids for activating ferroptosis in deep tumor sites, highlighting their potential for other therapeutic delivery and disease modulation applications.

## Graphical abstract

This study introduces a bacterial biohybrid (Ec@ZFOY) designed to target deep tumor regions using a combination of magnetic and hypoxia‐driven mechanisms for enhanced therapeutic delivery. By incorporating hypoxia‐targeted Escherichia coli and magnetic nanoparticles loaded with a HIF‐1α inhibitor, the biohybrid activates ferroptosis through reactive oxygen species generation and glutathione depletion. The findings highlight the potential of this dual‐targeted system for effective cancer treatment and broader therapeutic applications.

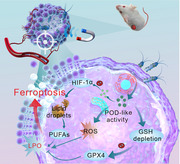

## 1 Introduction

Living cells can be engineered into innovative therapeutics that dynamically respond to external and environmental cues, enabling precise and adaptive therapeutic interventions [1, 2]. Various types of living cells have been developed for use as drugs or drug delivery systems in tumor therapy, including chimeric antigen receptor T cells, neutrophil, platelet, microphage, algae, yeast, and bacteria [3, 4, 5]. Among these, bacteria have garnered significant attention due to their easy of large‐scale cultivation, effective designability, convenient functionalization, and intrinsic tumor targeting capabilities [6, 7, 8, 9, 10, 11, 12, 13, 14]. For instance, subtypes of *Escherichia coli* (*E. coli*), such as MG1655, can target tumors by responding to hypoxia and chemical cues in the tumor microenvironment [15, 16]. Additionally, hypoxia‐tropic bacteria tend to accumulate in the deeper regions of tumors, which exhibit more severe hypoxic conditions than superficial layers [8, 17]. Cells in these deeper tumor sites display elevated proliferation, heterogeneity, and metastatic potential, making them crucial targets for drug delivery to enhance therapeutic efficacy [18, 19, 20, 21, 22]. Therefore, bacteria have been widely utilized to improve the tumor‐targeting capabilities of small therapeutics and nanosized particles, achieving superior tumor treatment outcomes [23, 24, 25, 26]. Furthermore, clinical studies have provided compelling evidence supporting the therapeutic potential of bacterial‐based approaches in cancer treatment [27, 28].

However, tumor hypoxia presents a significant challenge to various treatments, including chemotherapy, radiotherapy, and photodynamic therapy [29, 30]. Furthermore, hypoxia promotes resistance to apoptosis and impairs multiple non‐apoptotic cell death pathways, such as cuproptosis and ferroptosis [31, 32, 33]. Ferroptosis, a form of programmed cell death triggered by iron and excessive lipid peroxidation (LPO), shows significant potential in tumor therapy [34, 35, 36, 37, 38, 39]. Hypoxia hampers the oxygenation of free polyunsaturated fatty acids (PUFAs), inhibiting LPO and ferroptosis [40]. Additionally, hypoxia activates the hypoxia‐inducible factor 1α (HIF‐1α) signaling pathway and elevates the biogenesis of endogenous lipid droplets by facilitating the uptake of fatty acids and their storage [41, 42]. This process inhibits ferroptosis by sequestering oxidation‐sensitive PUFAs and preventing subsequent lipid peroxidation [43].

Although nanoparticles and living cells have been employed to enhance the pharmacokinetics and tumor targeting of ferroptosis inducers, their therapeutic efficacy is undermined by tumoral hypoxia, especial in the deeper regions of tumors. Moreover, strategies such as tumor‐specific catalysis and O_2_‐contained modality delivery can promote ferroptosis by alleviating hypoxia [44, 45, 46, 47, 48, 49, 50]. However, delivering therapeutics to tumor deep sites with high efficiency remains challenging [51], compromising their capacity for ferroptosis activation and therapeutic efficacy. Therefore, designing platforms for relieving tumor hypoxia and activating ferroptosis in deeper regions of tumors remains highly desirable and challenging.

**SCHEME 1:**
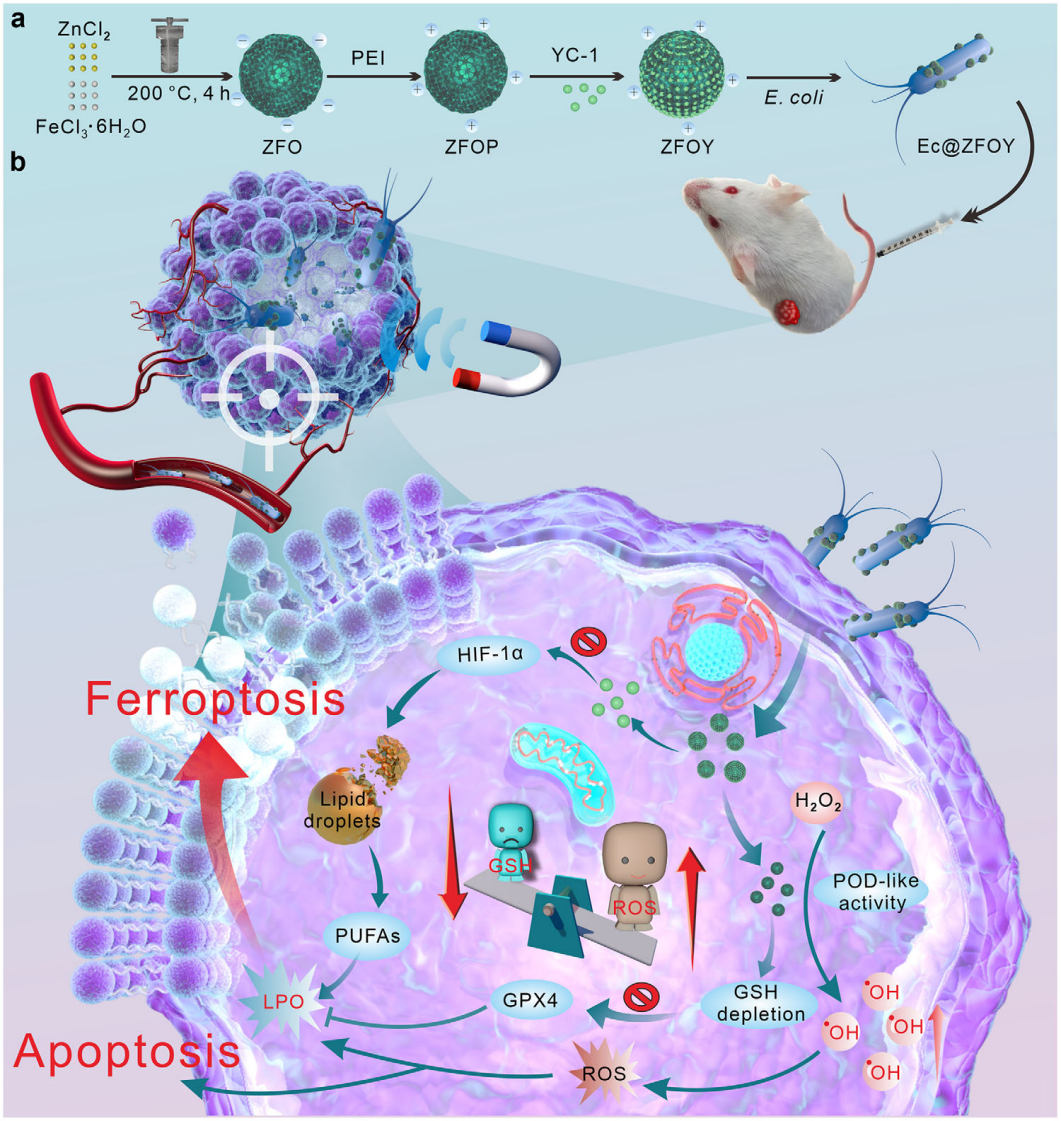
Schematic representation of the synthesis and therapeutic process of Ec@ZFOY biohybrids. (a) Schematic illustration of Ec@ZFOY biohybrid synthesis. (b) Antitumor mechanism of Ec@ZFOY biohybrids.

Considering these challenges, we hypothesize that a living bacterial biohybrid can be designed to dynamically respond to external and environmental cues [52, 53], enabling deep tumor targeting and enhanced ferroptosis. In this study, we introduce a bacterial biohybrid, termed Ec@ZFOY, which consists of hypoxia‐targeted *E. coli* (MG1655, Ec) and porous magnetic Zn_0.16_Fe_1.24_O_4_ (ZFO) nanoparticles loaded with Lificiguat (YC‐1), a HIF‐1α inhibitor, to activate tumor ferroptosis (Scheme 1a). The ZFO endows Ec@ZFOY with magnetic‐steering properties in addition to its intrinsic hypoxia‐responsive capability, leading to highly efficient tumor accumulation and deep tumor penetration under magnetic guidance. This dual targeting strategy enhances therapeutic efficacy while minimizing off‐target effects, simultaneously.

*E. coli's* hypoxia‐seeking capability facilitates autonomous navigation through the dense stromal matrix, ensuring precise delivery of nanomaterials to hypoxic tumor regions. ZFO nanoparticles exhibit (POD)‐ and glutathione (GSH) oxidase (GSHox)‐like activities, increasing oxidative stress by catalyzing tumoral H_2_O_2_ into hydroxyl radical (^•^OH) and suppressing glutathione peroxidase 4 (GPX4) by depleting GSH, respectively, thereby inducing cell ferroptosis. Furthermore, ZFO nanoparticles can release YC‐1 in the acidic tumor microenvironment, leading to HIF‐1α inhibition and subsequent reduction of lipid droplets. This modulation of the HIF‐1α‐lipid axis by the Ec@ZFOY bacterial biohybrid results in the release of PUFAs, further enhancing ferroptosis (Scheme 1b). In a breast tumor mouse model, Ec@ZFOY demonstrate a high tumor inhibition rate and minimal side effects, showing promise for future tumor treatments.

## 2 Results and Discussions

### 2.1 Fabrication and Characterizations of ZFO

**FIGURE 1:**
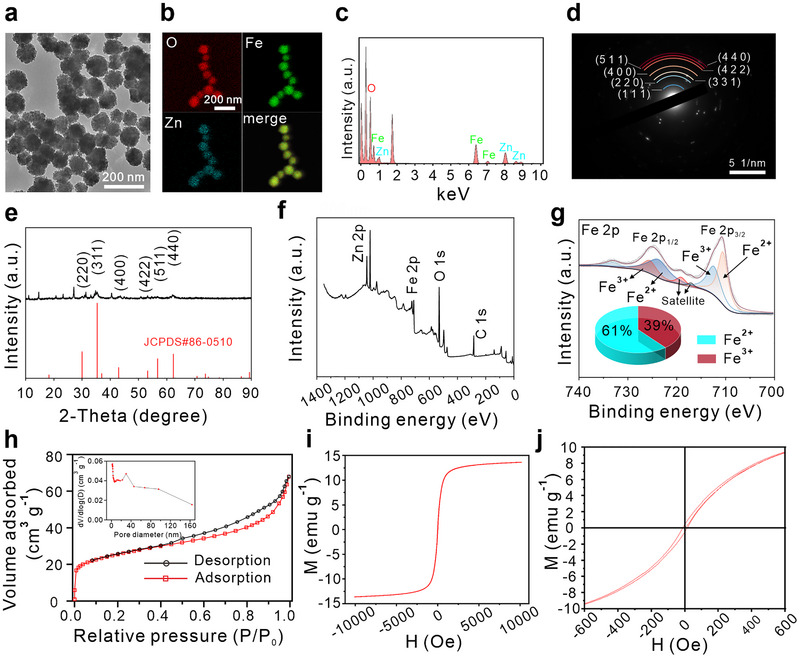
Composition and characterization of materials. (a) TEM image of ZFO. (b) Elemental mapping of O, Fe, and Zn in ZFO. (c) Total surface spectrogram. (d) HRTEM images with SAED patterns. (e) XRD patterns. (f,g) XPS spectra (f) and Fe 2p XPS spectra (g) of ZFO. (h) N_2_ adsorption−desorption isotherms and pore size distributions of ZFO. (i, j) Magnetic performance of ZFO measured by VSM at room temperature.

We synthesized ZFO nanoparticles using a hydrothermal method by mixing various ratios of ZnCl_2_ and FeCl_3_ in an ethylene glycol solution at high temperature (see experimental section). As shown in Figure 1a and Figure S1, Supporting Information, sizes of ZFO‐1 (ZnCl_2_: FeCl_3_ = 1:1.49), ZFO‐2 (ZnCl_2_: FeCl_3_ = 1:1.33), and ZFO‐3 (ZnCl_2_: FeCl_3_ = 1:1.05) were 100, 100, and 500 nm, respectively, with ZFO exhibiting a uniform morphology. Then, we evaluated their catalytic potential using a 3,3',5,5'‐tetramethylbenzidine (TMB) oxidative assay [54]. As depicted in Figure S2, Supporting Information, ZFO‐1 showed the highest absorbance at 652 nm in the presence of H_2_O_2_, indicating superior POD‐like activity and tumor‐specific ^•^OH generation. Therefore, we selected the ZFO‐1 (donated as ZFO) in the subsequent experiments due to its optimal size, uniform morphology, and superior POD‐like activity. Dynamic light scattering (DLS) analysis revealed a hydrodynamic diameter of approximately 220 nm for ZFO (Figure S3, Supporting Information). The larger particle size observed from DLS measurement than that from TEM is generally attributed to the formation of hydration layers on the particle surface. Energy‐dispersive X‐ray spectroscopy (EDS) element mapping images demonstrated the homogeneous distribution of oxygen (O), iron (Fe), and zinc (Zn) (Figure 1b). The atomic percentages in the overall surface spectrum confirmed the composition as Zn_0.16_Fe_1.24_O_4_ (Figure 1c). Selected area electron diffraction (SAED) patterns (Figure 1d) and X‐ray diffraction (XRD) results (Figure 1e) indicated that ZFO belongs to the cubic (Zn_0.35_Fe_0.65_)Fe_2_O_4_ phase (JCPDS No. 86–0510).

X‐ray photoelectron spectroscopy (XPS) analysis corroborated the presence of Zn, Fe, and O, consistent with the findings of EDS element mapping (Figure 1f). High resolution XPS spectra of Fe 2p revealed two binding energy peaks at 710.5 and 723.7 eV, corresponding to the Fe^2+^ main peak, and peaks at 712.4 and 725.5 eV corresponding to the Fe^3+^ main peak, confirming the coexistence of Fe^3+^ and Fe^2+^ in ZFO nanoparticles (Figure 1g). Additionally, XPS analysis indicated an Fe^3+^ to Fe^2+^ ratio of 39:61.

The N_2_ isothermal adsorption–desorption curve reveals a specific surface area of 91.65 m^2^ g^−1^ and an average pore diameter of 6.7345 nm (Figure 1h). This large specific surface area and porous structure indicate a strong adsorption capacity, which is advantageous for drug loading. Additionally, Figure S4, Supporting Information, demonstrates the magnetic attraction of ZFO nanoparticles. To further evaluate these magnetic properties, we used a vibrating sample magnetometer (VSM) at room temperature. Figure 1i,j displays a narrow, steep hysteresis loop with low coercivity, characterization of a soft magnetic material. The saturation magnetization (MS) was measured at 13.6 emu g^−1^. The magnetic saturation intensity of ZFO is slightly lower than that of Fe_3_O_4_, primarily attributing to the nanoscale size of ZFO and the incorporation of Zn^2^^+^ ions into its structure (Figure S5, Supporting Information) [55].

**FIGURE 2:**
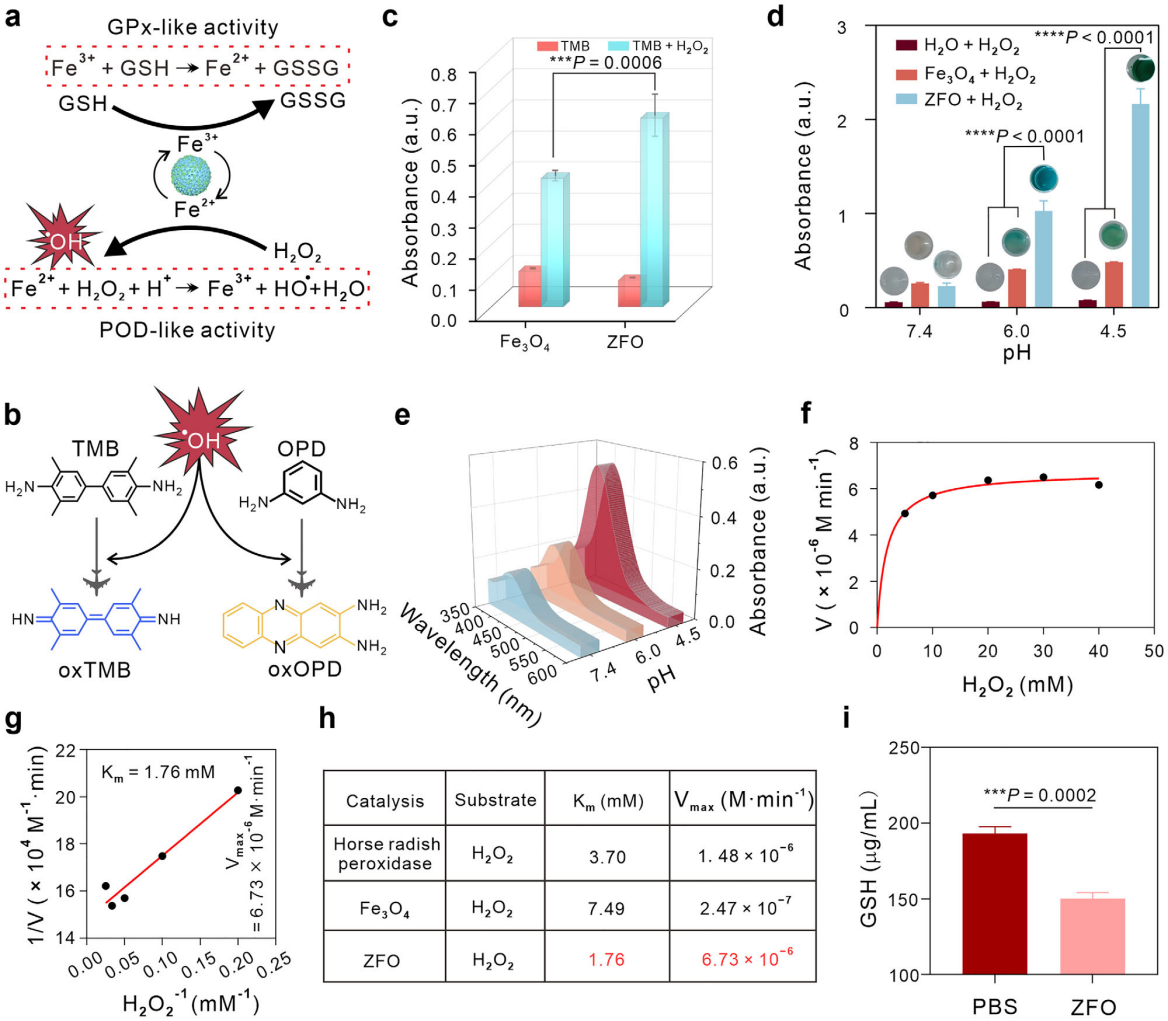
Enzyme‐mimicking properties of ZFO nanoparticles. (a) Diagram of enzyme‐like reaction of ZFO. (b) Schematic representation of the chromogenic reaction of ^•^OH with TMB and OPD. (c) Comparison of TMB oxidation of ZFO and commercial Fe_3_O_4_ nanoparticles at pH 6.0. [H_2_O_2_] = 75 µM. (d) Absorbance changes of TMB treated with PBS, Fe_3_O_4_, and ZFO at different pH values in the presence of H_2_O_2_ (75 µM). (e) Detection of ^•^OH by OPD at different pH values. (f,g) Michaelis−Menten kinetic analysis and Lineweaver–Burk plot for ZFO using H_2_O_2_ as a substrate. (h) Comparison of kinetic parameters of POD‐mimicking activity. (i) GSHOx‐like activity of ZFO. Statistical significance denoted as ***p < 0.001 and ****p < 0.0001. Data in (d) are analyzed by one‐way ANOVA, followed by Tukey's multiple comparison tests. Data in (i) are analyzed by two tailed *t* test. Data represent mean ± s.d.

XPS analysis reveals the presence of both Fe^3^^+^ and Fe^2^^+^ in ZFO, indicating its capacity to deplete GSH and generate ^•^OH [56]. Next, we further investigated the GSHox‐ and POD‐mimicking activates of ZFO nanoparticles (Figure 2a,b). As shown in Figure 2c,d, the POD‐like activity of ZFO surpasses that of commercialized Fe_3_O_4_ nanoparticles, with catalytic efficiency increasing as the solution pH decreases. We further assessed ZFO's POD‐like activity using *o*‐phenylenediamine (OPD), which exhibits maximal absorption at 425 nm [57, 58]. The UV–vis absorbance spectra demonstrate increased absorption at pH 4.5, indicating enhanced ^•^OH production (Figure 2e). Subsequently, we evaluated ZFO's catalytic activity by varying H_2_O_2_ concentrations as reaction substrates, enabling a comprehensive assessment through steady‐state catalytic kinetics. Figure 2f,g and Figure S6, Supporting Information, illustrate the Michaelis–Menten equation and Lineweaver–Burk plots, showing that ZFO has a *K*_m_ value of 1.76 mM and a maximum reaction rate (*V*_max_) of 6.73 × 10^−6^ M min^−1^. These values are superior to those of Fe_3_O_4_, which has *K*_m_ and *V*_max_ values of 7.49 mM and 2.47 × 10^−7^ M min^−1^, respectively (Figures S7–S9). Horseradish peroxidase, a standard enzyme with high affinity for H_2_O_2_, was utilized to benchmark the POD‐like activity of the nanozymes. Figure 2h reveals that ZFO exhibit substantially higher *V*_max_ values than horseradish peroxidase, underscoring its superior enzyme‐like activity. Particularly, ZFO displayed a superior *V*_max_ and lower *K*_m_ compared to Fe_3_O_4_, highlighting its remarkable POD‐like activity. Figure 2i and Figure S10, Supporting Information, show a decrease in GSH concentration following the co‐incubation of ZFO with GSH, indicating that ZFO exhibits GSHox properties and has the capacity to deplete GSH.

### 2.2 Characterization of Ec@ZFOY Conjugates

**FIGURE 3:**
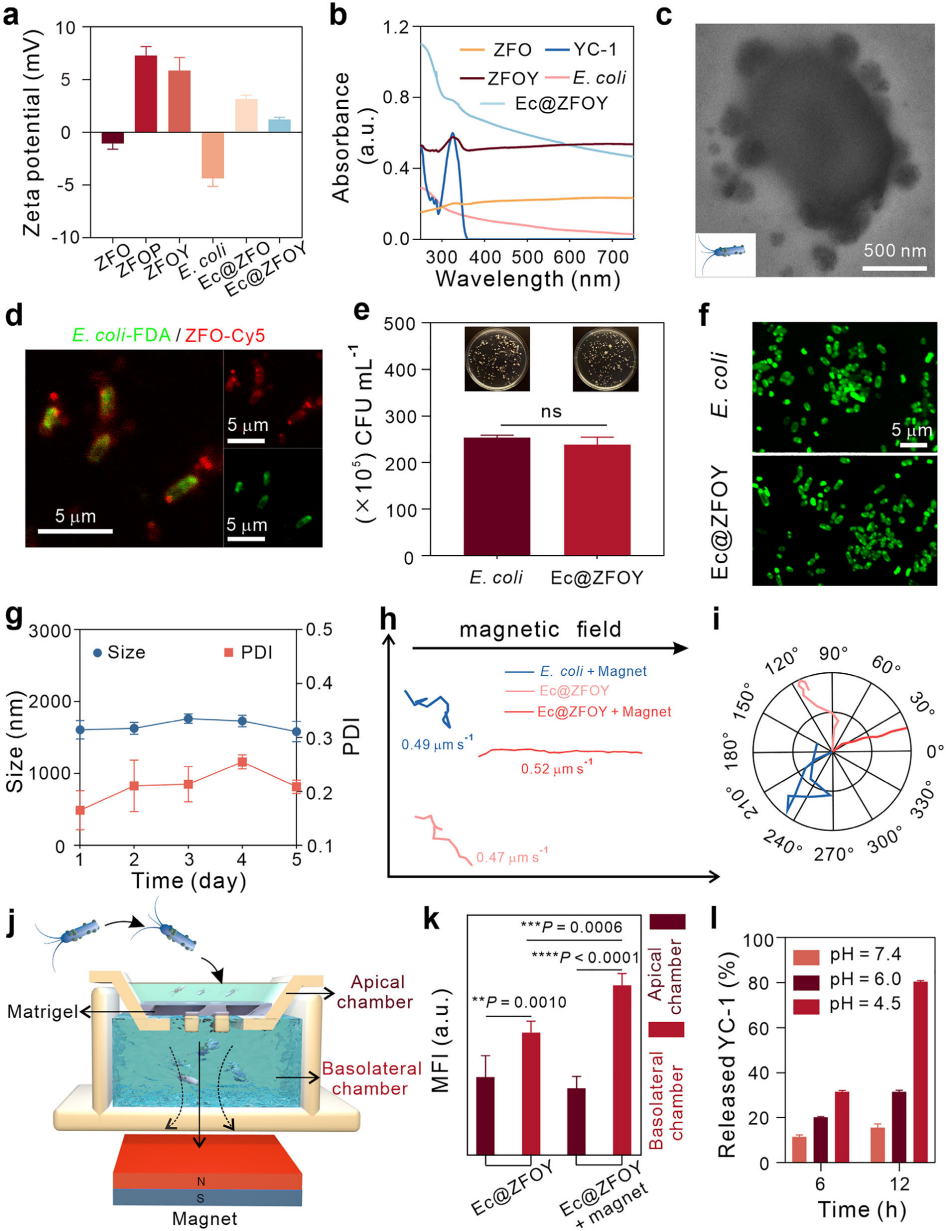
Characterization of Ec@ZFOY conjugates. (a) Zeta potential measurements of various formulations. (b) UV–vis absorption spectra of different formulations. (c) TEM image of Ec@ZFOY. (d) Fluorescence co‐localization observed using LSCM: fluorescein diacetate‐labeled *E. coli* (green) and Cy5‐labeled ZFO (red). (e) Proliferative capacity of bacterial biohybrids assessed using the plate coating method. (f) Bacterial biohybrid activity detected by fluorescein diacetate staining. (g) Long‐term hydrodynamic diameter of Ec@ZFOY (*n* = 3). (h,i) Trajectories (h) and polar coordinates (i) of different objects marked by fluorescein diacetate observed by LSCM. (j) Schematic diagram of the transwell experiment. (k) Quantitative analysis of fluorescence intensity in the transwell assay to evaluate the penetration ability of bacterial biohybrids. (l) The release of YC‐1 from ZFOY in various conditions. Statistical significance denoted as **p < 0.01, ***p < 0.001, ****p < 0.0001, and ns: not significant (*p* > 0.05). Data in (e) are analyzed by two tailed *t* test. Data in (k) are analyzed by one‐way ANOVA, followed by Tukey's multiple comparison tests. Data represent mean ± S.D.

Based on the zeta potential measurements, both ZFO and *E. coli* exhibit similar electric charge, hindering their combination (Figure 3a). Hence, ZFO was modified with polyethyleneimine (PEI) to modify its charge (ZFOP). Subsequently, YC‐1 small molecule drugs were loaded onto ZFOP to form ZFOY NPs. Finally, *E. coli* was co‐incubated with ZFOY to form Ec@ZFOY conjugates. The UV/Vis–NIR absorption spectra and zeta potential confirmed the successful preparation of YC‐1‐loaded biohybrids (Figure 3a,b). The amount of YC‐1 was quantitatively analyzed using a standard curve, resulting in a loading efficiency of 51.7% (Figure S11, Supporting Information). TEM images reveal abundant ZFOY conjugated to *E. coli* (Figure 3c and Figure S12, Supporting Information), indicating the successful preparation of the biohybrids. Additionally, we used atomic absorption spectroscopy to measure the iron (Fe) concentration in both ZFO and Ec@ZFOY samples. The results indicated that *E. coli* achieved a loading efficiency of 80.5% (Figure S13, Supporting Information). Fluorescein diacetate, a cell‐permeable esterase substrate, was utilized for bacterial labeling and viability assessment. As shown in confocal laser scanning microscopy (CLSM, Sunny Optical Technology, SOPTOP CLSM610, China) images (Figure 3d), the red fluorescence of Cy5‐labelled ZFO nanoparticles colocalizes with the green fluorescence emitted by fluorescein diacetate‐labelled *E. coli*, further confirming the successful conjugation of nanoparticles to *E. coli*. The motility of bacteria influences their distribution and accumulation within tumors. Bacterial activity was assessed using the dilution coating plate method. Equal concentrations of Ec@ZFOY and *E. coli* were diluted and evenly spread on agar plates. Following incubation at 37°C for 12 h, the bacterial colonies were enumerated.

Subsequently, the bacterial count was adjusted based on the dilution factor and the volume inoculated. As can be seen from Figure 3e, the colony numbers did not significantly differ between these two groups, indicating that the nanoparticles had no adverse effects on the activity of *E. coli*. Following incubation with fluorescein diacetate, both *E. coli* and Ec@ZFOY exhibited evident green fluorescence, proving that the bacterial activity remained unaffected (Figure 3f). Hydrodynamic diameters of *E. coli*, ZFOY, and Ec@ZFOY were 1260 (Figure S14, Supporting Information), 311 (Figure S15, Supporting Information), and 1513 nm (Figure S16, Supporting Information), respectively. The increased diameters of Ec@ZFOY compared to primary *E. coli* further confirmed the successful preparation of Ec@ZFOY. Long‐term dynamic light scattering analysis of Ec@ZFOY revealed that the size of the bacterial biohybrid conjugate remained unchanged over time in PBS buffer, confirming its structural stability (Figure 3g). Additionally, the polymer dispersibility index exhibited no significant change, consistently remaining below 0.3, indicative of excellent dispersibility. The hydrodynamic size of Ec@ZFOY exhibited stable during storage in FBS within 5 days, indicating the excellent stability and dispersibility of Ec@ZFOY (Figure S17, Supporting Information).

Decidedly, *E. coli* dose not exhibit magnetic properties (Figure S18a, Supporting Information). In contrast, Ec@ZFOY migrates toward a magnet (Figure S18b, Supporting Information), demonstrating its magnetism. We then evaluated the motion trajectories of *E. coli* and Ec@ZFOY using CLSM. As shown in Figure 3h, *E. coli* displayed irregular motion with a speed of 0.49 µm s^−1^ under magnetic steering. In the absence of a magnetic field, Ec@ZFOY also exhibited random motion at a speed of 0.47 µm s^−1^, indicating that the ZFOY attachment dose not impair the bacteria's free movement. Notably, under magnetic guidance, Ec@ZFOY moved directionally aligned with the magnetic field at a slightly higher speed of 0.52 µm s^−1^ compared to the other groups, highlighting its magnetic tropism (Figure 3i). While the variation in motion speed is minimal, the displacement (defined as the maximum distance traveled in a particular direction) showed a marked difference: 28.80 µm for Ec@ZFOY and 121.95 µm for Ec@ZFOY with magnetic guidance over 5 min, clearly indicating magnetic targeting‐driven directional migration (Table S1, Supporting Information). Furthermore, we assessed the penetration and move directional movement of Ec@ZFOY using a transwell assay (Figure 3j). A Matrigel layer was applied to the bottom of the upper chamber, and fluorescein diacetate‐labelled Ec@ZFOY was added to the upper chamber. A magnet was placed beneath the lower chamber to generate a magnetic field, and the setup was incubated at 37°C for 12 h. As shown in Figure 3k, the fluorescence intensity in the lower layer of the magnet group was stronger than that of the control group. This indicates that the magnet effectively guided Ec@ZFOY through the matrix layer, allowing it to migrate from the top chamber to the bottom chamber, thus confirming the bacterial biohybrid's penetration ability and magnetic propulsion. Additionally, ZFOY nanoparticles released YC‐1 with a pH‐responsive manner, suggesting that Ec@ZFOY has a tumor microenvironment‐responsive therapeutic release capability (Figure 3l). In summary, these results demonstrate that Ec@ZFOY, with its stable structure, exhibits directional movement under magnetic field control while maintaining bacterial functionality. This suggests significant potential for further applications of the hybrid bacterial biohybrid.

### 2.3 Evaluation of the Anti‐Tumor Effect of Ec@ZFOY Conjugates In Vitro

**FIGURE 4:**
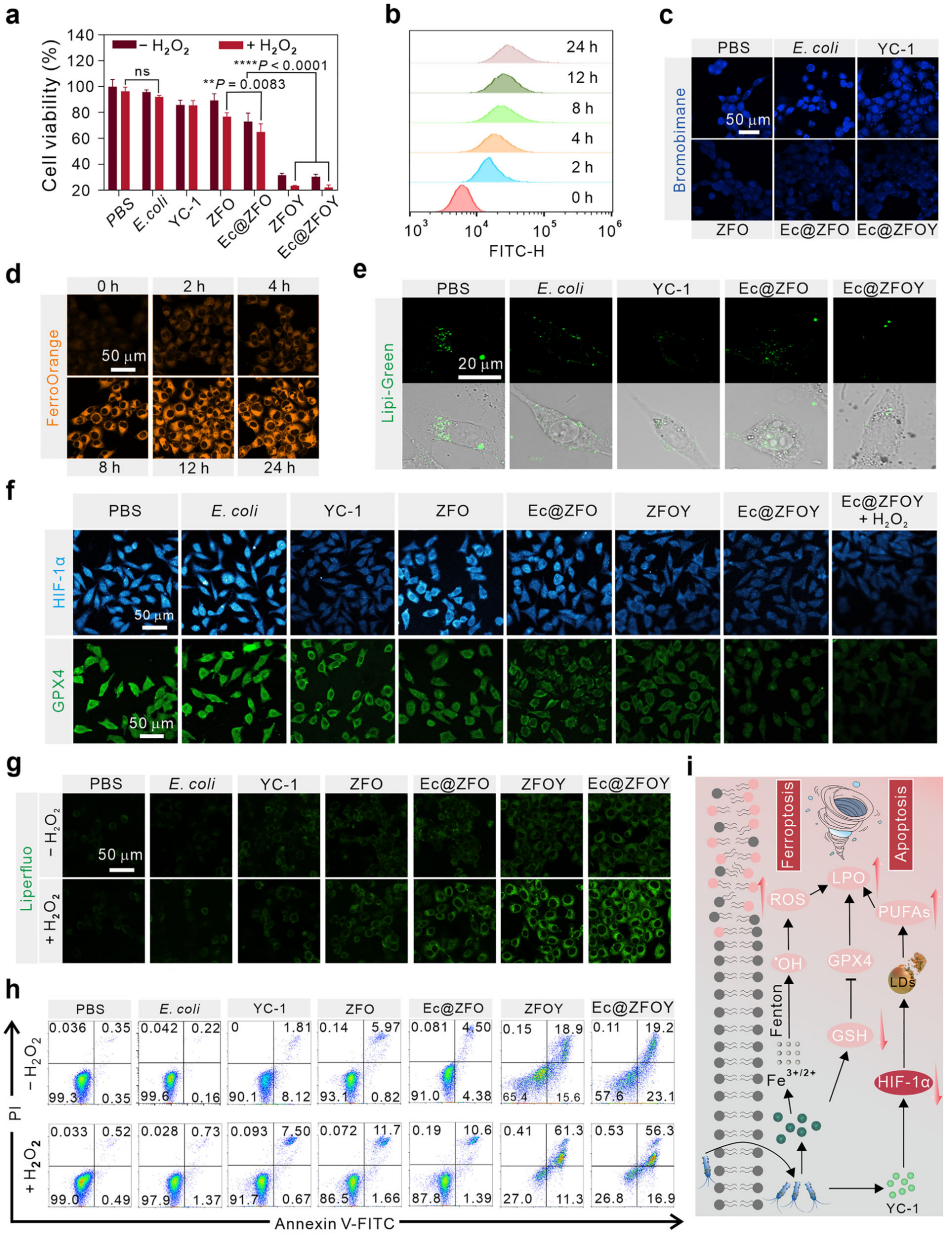
In vitro evaluation of Ec@ZFOY‐mediated apoptosis and ferroptosis in 4T1 cells. (a) Cell viability of 4T1 cells treated with different samples with or without H**_2_** O**_2_** (*n* = 6) (b) In vitro flow cytometric ZFO phagocytosis assay after varied durations of incubation. (c) Intracellular GSH depletion after different treatments. (d) Fluorescence images of FerroOrange in 4T1 cells after different incubation time. (e) Detection of lipid droplets of 4T1 cells after different treatments stained by Lipi‐Green. (f) Immunofluorescence staining of HIF‐1α and GPX4 in 4T1 cells. (g) LPO assay of the treated 4T1 cells using Liperfluo probe. (h) Flow cytometric apoptosis assay of 4T1 cells after various treatments. (i) Schematic illustration of Ec@ZFOY for evoking ferroptosis and apoptosis. Statistical significance denoted as **p < 0.01, ****p < 0.0001, and ns: not significant (*p* > 0.05). Data in (a) are analyzed by one‐way ANOVA, followed by Tukey's multiple comparison tests. Data represent mean ± SD.

Firstly, we evaluated the cytotoxicity of ZFO nanoparticles and YC‐1 using a CCK‐8 assay in 4T1 cells. As shown in Figures S19 and S20, both ZFO and YC‐1 exhibited concentration‐dependent therapeutic effects on cancer cells. We then investigated their synergistic effects on cell viability (Figure 4a). Treatment with ZFO (40 µg mL^−1^) and YC‐1 (2 µg mL^−1^) resulted in cell viabilities of 89% and 85%, respectively. In contrast, ZFOY and Ec@ZFOY reduced cell viability to 30%, and further to about 20% in the presence of H_2_O_2_. Additionally, we assessed the impact of ZFO on normal cells using NIH/3T3 cells. The results showed no cell death across all groups treated with various concentrations of Ec@ZFOY, indicating its specific cytotoxicity towards tumor cells (Figure S21, Supporting Information). The in vitro therapeutic effects further investigated using live/dead staining (Figure S22, Supporting Information), where 4T1 cells treated with Ec@ZFOY in the presence of H_2_O_2_ showed significant red fluorescence, indicating cell death. These results demonstrate that YC‐1 enhances the therapeutic efficacy of ZFOY, and that Ec@ZFOY exhibits notable efficiency in cancer cell killing the presence of H_2_O_2_.

Subsequently, we studied the mechanisms of Ec@ZFOY in tumor treatment. We first analyzed the cellular uptake of fluorescein isothiocyanate (FITC)‐labeled ZFO‐anchored *E. coli* using flow cytometry. As depicted in Figure 4b, the fluorescence intensity of FITC increased with incubation time, indicating successful internalization of the bacterial biohybrid by cancer cells. Additionally, cellular GSH levels were evaluated by bromobimane staining (Figure 4c). Strong bule fluorescence signals in cells treated with PBS, *E. coli*, and YC‐1 indicated no decrease in cellular GSH. In contrast, cells incubated with ZFO, Ec@ZFO, and Ec@ZFOY exhibited weaker fluorescence signals compared to the PBS group, indicating GSH depletion and demonstrating the GSHox‐like activities of the ZFO nanoparticles. Accumulation of Fe^2+^ ions, which can specifically increase oxidative stress, is a hallmark of ferroptosis. We investigated cellular Fe^2+^ levels using FerroOrange fluorescent dye after Ec@ZFOY treatment. As shown in Figure 4d, cells displayed a time‐dependent increase in fluorescence intensity, suggesting that Ec@ZFOY can increase cellular Fe^2+^ concentration.

BBoxiProbe O26 staining. As can be seen from LSCM images (Figure S23, Supporting Information), Ec@ZFOY significantly increased the ^•^OH generation in the presence of H_2_O_2_. Furthermore, YC‐1‐loaded Ec@ZFOY significantly depleted cellular lipid droplets (Figure 4e). Figure 4f shows that YC‐1 endows Ec@ZFOY with the ability to downregulate the expression of HIF‐1α protein, resulting in hypoxia reversal and lipid droplet depletion. It was observed that ZFOY and Ec@ZFOY markedly suppressed the expression of GPX4, a key ferroptosis inhibitory protein, thus activating ferroptosis. Intracellular LPO levels were assessed using a fluorescent probe, Liperfluo (Figure 4g). In the absence of H_2_O_2_, ZFOY‐treated cells exhibited higher fluorescence signals than those treated with YC‐1 and ZFO, indicating their synergistic efficacy for lipid peroxidation and ferroptosis activation. Notably, in the presence of H_2_O_2_, LPO generation was significantly enhanced in the ZFO, Ec@ZFO, ZFOY, and Ec@ZFOY groups. These findings demonstrate that modulation of the HIF‐1α‐lipid pathway enables ferroptosis and that this bacterial biohybrid shows potential in inducing self‐enhanced ferroptosis in the tumor microenvironment. As shown in Figure 4h, Ec@ZFOY also triggered cell apoptosis due to induced cellular oxidative stress. Consequently, the prepared bacterial biohybrid induces cell death efficiently through synergistic apoptosis and ferroptosis by alleviating cellular oxidative stress and modulating the HIF‐1α‐lipid pathway (Figure 4i).

### 2.4 Penetration and In Vivo Biodistribution of Ec@ZFOY Bacterial Biohybrid

**FIGURE 5:**
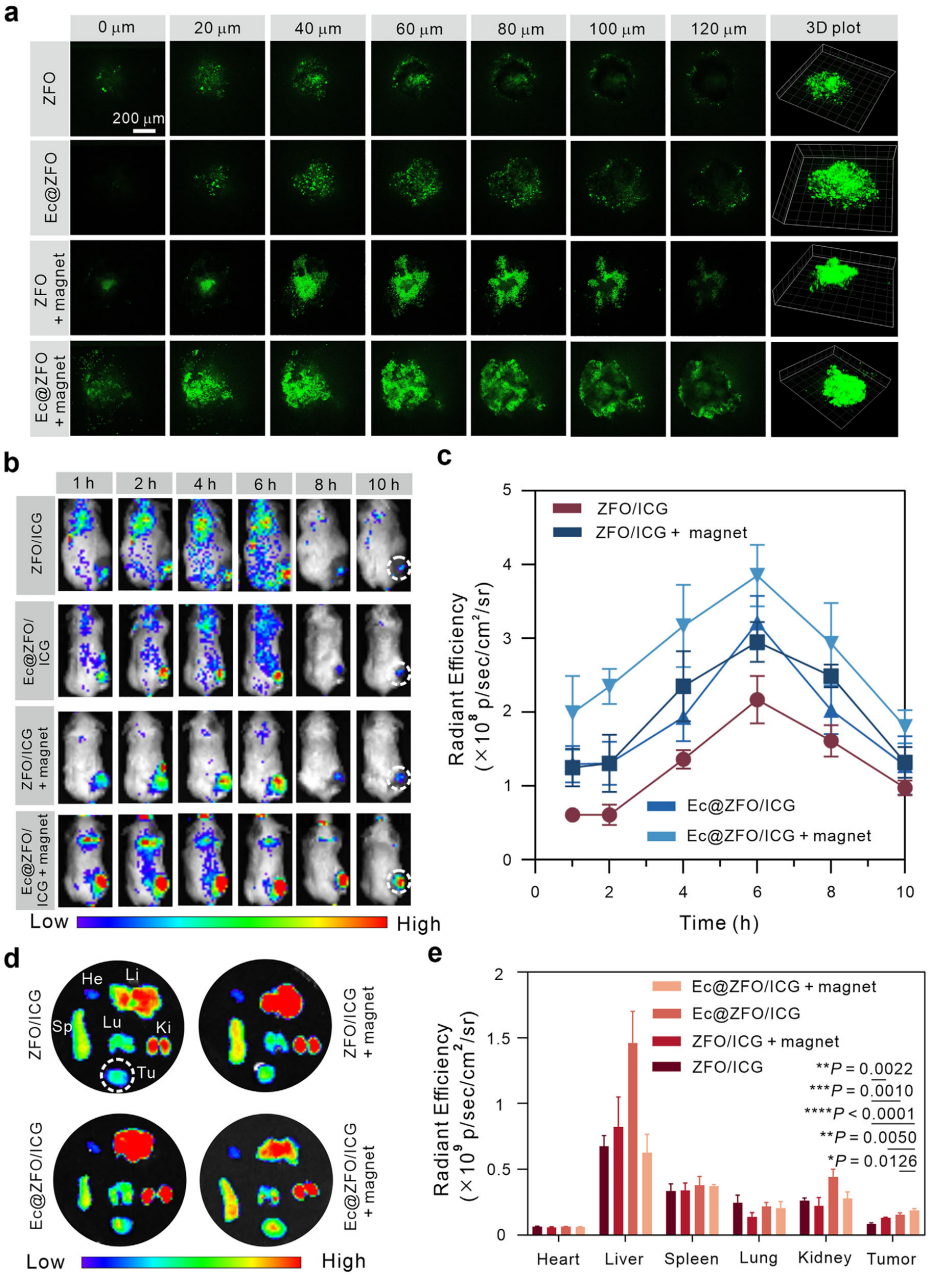
Tumor penetration and biodistribution of Ec@ZFOY. (a) *Z*‐axis scanned images of FITC signal in 3D spheroids treated with ZFO, Ec@ZFO, ZFO + magnet, and Ec@ZFO + magnet. (b) In vivo images of mice treated with ZFO/ICG, Ec@ZFO/ICG, ZFO/ICG + magnet, and Ec@ZFO/ICG + magnet at 1, 4, 6, 8, and 10 h post‐injection. (c) Quantification of the fluorescence intensities in in vivo images of mice. (d) Ex vivo images of the major organs and tumors harvested at 24 h post‐injection. He: heart, Li: liver, Sp: spleen, Lu: lung, Ki: kidney, Tu: tumor. (e) Quantification of the fluorescence intensities of the major organs and tumors. ns: not significant (*p* > 0.05), *p < 0.05, **p < 0.01, ***p < 0.001, and ****p < 0.0001, analyzed by one‐way ANOVA, followed by Tukey's multiple comparison tests. Data represent mean ± SD.

To investigate the penetration capabilities of the Ec@ZFOY bacterial biohybrid, we used cellular 3D spheroids. We labeled ZFO nanoparticles with fluorescein isothiocyanate (FITC) to track their localization within 3D tumor spheroids. The labeling efficiency was determined to be 86.5% (Figure S24, Supporting Information). Briefly, 4T1 multicellular spheroids were incubated with ZFO/FITC and Ec@ZFO/FITC for 12 h, without or with magnet guidance. Fluorescence images were captured by CLSM, using the loaded FITC as an indicator. As shown in Figure 5a, ZFO accumulated primarily at the superficial layers of the multicellular spheroids. However, its penetration was enhanced under magnet guidance. Without magnet steering, Ec@ZFO exhibited better tumor penetration compared to ZFO, attributed to the hypoxia‐targeting capacity of *E. coli* (MG1655). Notably, Ec@ZFO under magnet guidance demonstrated significantly higher fluorescence signals in the deeper regions of the multicellular spheroids than without magnet, indicating that Ec@ZFO possesses intrinsic tumor penetration ability, which is further enhanced by magnet steering. Subsequently, we investigated the tumor‐targeting capability of bacterial biohybrids through in vivo fluorescence imaging using ICG as a fluorescent indicator. The labeling efficiency of ICG was determined to be 88% (Figure S25, Supporting Information). By comparing the fluorescence intensity of ICG alone and ZFO/ICG, we confirmed that the presence of ZFO does not interfere with the fluorescence properties of the labeled molecules (Figure S26, Supporting Information). Twelve tumor‐bearing mice were divided into four groups and subjected to various treatments: ZFO/ICG, Ec@ZFO/ICG, ZFO/ICG + magnet, and Ec@ZFO/ICG + magnet. In vivo fluorescence images were captured using the IVIS imaging system from RWD Life science (MOIS HT Small Animal In Vivo Optical Imaging System, China) (Figure 5b,c). We found that tumor sites in the Ec@ZFO/ICG and ZFO/ICG + magnet groups displayed higher compared to the ZFO/ICG group, indicating that both *E. coli* and magnet guidance enhance the tumor‐targeting of the ZFO nanoparticles. Specifically, Ec@ZFO/ICG under magnet guidance exhibited the highest fluorescence signals in tumor sites.

After 24 h post‐injection, the mice were euthanized, and their tumors and major organs were collected and imaged. As illustrated in the ex vivo images (Figure 5d) and corresponding intensities (Figure 5e and Figure S27, Supporting Information), Ec@ZFO/ICG + magnet demonstrated superior tumor‐targeting capacities compared to other groups.

These findings underscore the significant potential of Ec@ZFOY for deep tumor penetration and targeted delivery, enhanced by magnetic guidance.

### 2.5 Evaluation of the Anti‐Tumor Effect of Ec@ZFOY In Vivo

**FIGURE 6:**
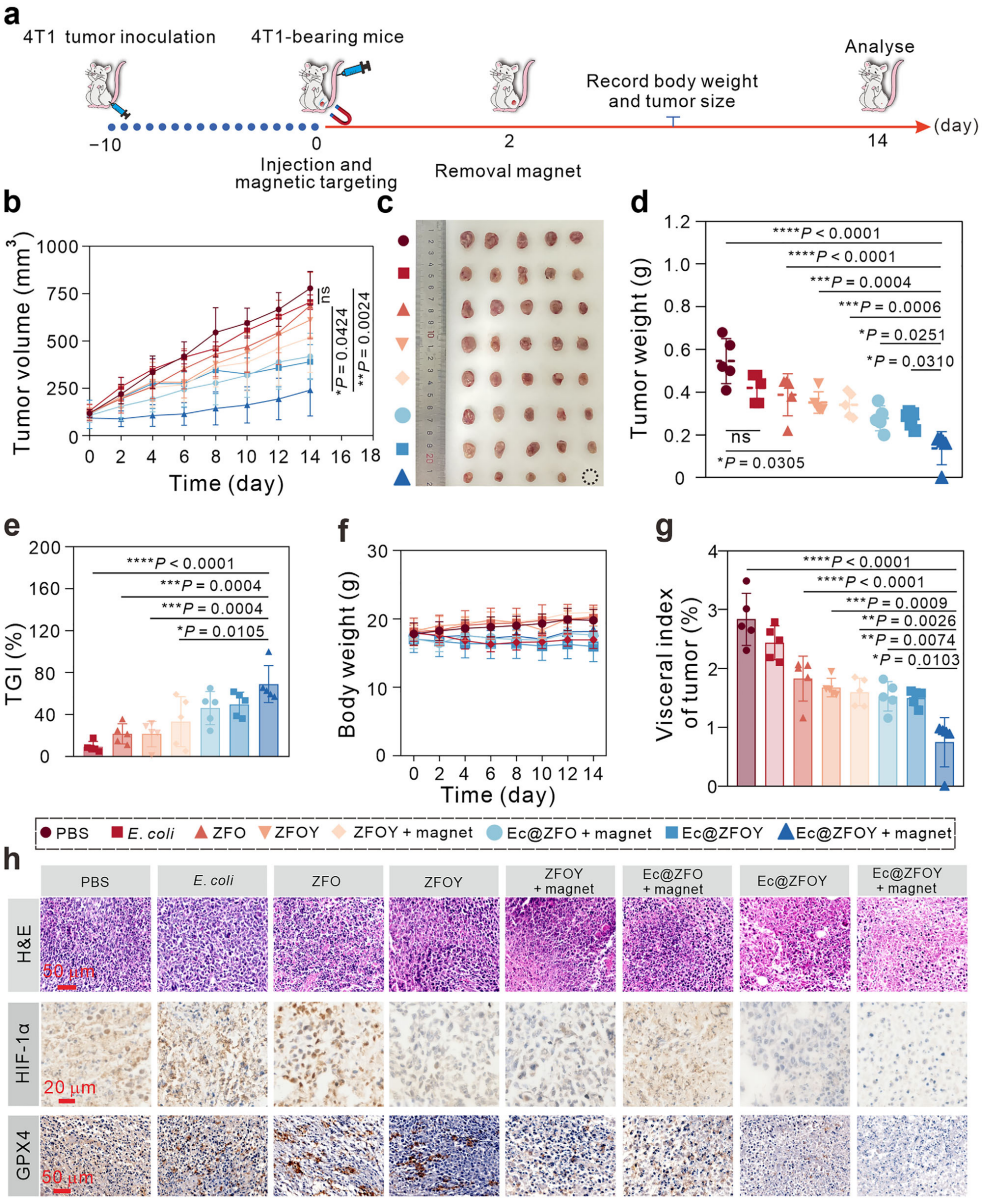
In vivo anti‐tumor activity of Ec@ZFOY. (a) Schematic timeline of the in vivo tumor treatment. (b) Tumor growth curves of following different treatments (*n* = 5). (c) Photographs of tumors from various groups after 14 days of therapy. (d) Tumor weight in mice subjected to different treatments (*n* = 5). (e) Tumor growth inhibition rates following different treatments (*n* = 5). (f) Body weights of 4T1 tumor‐bearing mice during 14‐day treatment (*n* = 5). (g) Tumor visceral index of mice treated with different formulations after 14 days. (h) H&E and immunohistochemistry analyses of tumor slices following different treatments. ns: not significant (*p* > 0.05), *p < 0.05, **p < 0.01, ***p < 0.001, and ****p < 0.0001, analyzed by one‐way ANOVA, followed by Tukey's multiple comparison tests. Data represent mean ± SD.

Subsequently, we evaluated the in vivo tumor therapeutic efficacy of Ec@ZFOY in 4T1 tumor‐bearing mice. Forty tumor‐bearing were randomly divided into eight groups (*n* = 5) and subjected to the following treatments: (i) PBS, (ii) *E. coli* (1 × 10^6^ CFU mL^−1^, 100 µL), (iii) ZFO (200 µg mL^−1^, 100 µL), (iv) ZFOY (200 µg mL^−1^, 100 µL), (v) ZFOY + magnet, (vi) Ec@ZFO + magnet, (vii) Ec@ZFOY, and (viii) Ec@ZFOY + magnet. On day 0, the mice were received intravenous injection of the assigned formulations (Figure 6a), and magnets were applied to the corresponding groups and removed after 2 days.

Throughout the 14‐day treatment period, body weights and tumor volume were monitored every 2 days. As shown in Figure 6b, tumors in the PBS and *E. coli* groups grew rapidly. ZFO and ZFOY showed moderate tumor growth inhibition, which was enhanced with magnet and *E. coli* guidance. Notably, Ec@ZFOY under magnet guidance significantly delayed tumor growth. After 14 days of treatments, the mice were euthanized, and their tumors were imaged (Figure 6c) and weighted (Figure 6d). The tumor growth inhibition (TGI) rate of Ec@ZFOY with magnet guidance was 69.1% (Figure 6e). Throughout the treatment, no significant changes in body weight were observed across all groups, indicating minimal side effects (Figure 6f). The visceral indices (tumor weight/body weight) were 2.84% for PBS, 2.44% for *E. coli*, 1.83% for ZFO, 1.68% for ZFOY, 1.60% for ZFOY + magnet, 1.53% for Ec@ZFO + magnet, 1.50% for Ec@ZFOY, and 0.75% for Ec@ZFOY + magnet, confirming the notable therapeutic efficacy of the bacterial biohybrid under magnet guidance (Figure 6g).

We also investigated the therapeutic mechanisms of the bacterial biohybrid in tumor inhibition. Hematoxylin and eosin (H&E) staining of tumor slices revealed that Ec@ZFOY under magnet guidance induced severe cell damage (Figure 6h). Immunohistochemical staining showed that Ec@ZFOY + magnet significantly suppressed the expression of HIF‐1α and GPX4, indicating hypoxia reversal and ferroptosis activation in tumors. These findings highlight the potential of promoting ferroptosis through precise therapeutic delivery and down‐regulation of HIF‐1α, demonstrating remarkable efficacy in cancer treatment.

### 2.6 Systematic In Vivo Biocompatibility Assay

**FIGURE 7:**
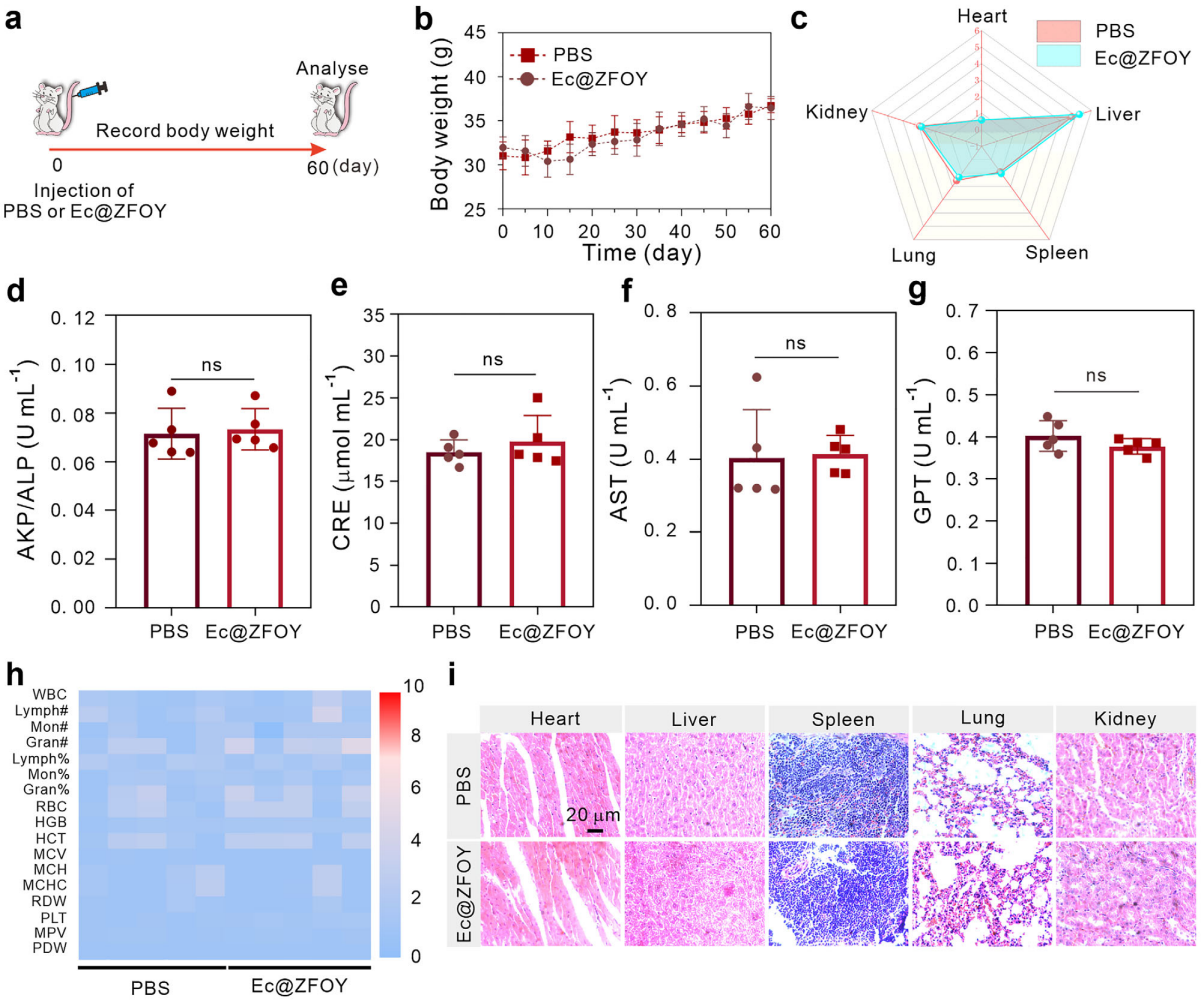
In vivo biosafety assessment. (a) Schematic diagram illustrating the evaluation of bacterial biohybrid biocompatibility. (b) Body weights of mice treated with PBS and Ec@ZFOY over a 60‐day period (*n* = 5). (c) Comparison of organ indices (organ weight/body weight) after treatments. (d–g) Serum biochemical parameters of AKP/ALP (d), CER (e), AST (f), and GPT (g). (h) Hematology analysis from mice treated with PBS and Ec@ZFOY (*n* = 5). (i) H&E staining images of major organs from mice treated with PBS and Ec@ZFOY (*n* = 5). Statistical significance of the groups was analyzed by Student's *t*‐test (two‐tailed). ns: not significant, p > 0.05. Data represent mean ± SD.

Finally, we evaluated the biocompatibility of the bacterial biohybrid Ec@ZFOY. First, we studied hemolytic effects of ZFO nanoparticles. As shown in Figure S28, Supporting Information, ZFO at a high concentration of 300 µg mL^−1^ exhibited negligible hemolysis rates in red blood cells, indicating good biocompatibility. We then investigated the long‐term toxicity of Ec@ZFOY in mice. Ten healthy mice were divided into two groups and treated with PBS or Ec@ZFOY via tail vein injection (Figure 7a). Mouse body weights were monitored every 5 days over a 60‐day period. As illustrated in Figure 7b, both groups showed consistent increases in body weights, indicating minimal side effects from the bacterial biohybrids. On day 60, the mice were euthanized, and their health‐related parameters were examined. As shown in Figure 7c, there were no significant differences in the indices of major organs (heart, liver, spleen, lung, and kidney) between the PBS and Ec@ZFOY groups. Additionally, Ec@ZFOY exhibited minimal effects on serum indices, including alkaline phosphatase (AKP/ALP, Figure 7d), creatinine (CRE, Figure 7e), glutamic oxalacetic transaminase (AST, Figure 7f), and glutamic‐pyruvic transaminase (GPT, Figure 7g), compared to the PBS groups. This indicates that Ec@ZFOY dose not affect murine hepatorenal functions. Furthermore, Ec@ZFOY did not influence murine blood parameters (Figure 7h) or induce tissue damage (Figure 7i). These findings demonstrate the favorable biocompatibility of Ec@ZFOY, supporting its potential for future applications in therapeutic delivery and disease modulation.

## 3 Conclusion

In summary, we have successfully engineered a bacterial biohybrid, Ec@ZFOY, that exhibits dual magnetic and hypoxia‐tropic properties for efficient therapeutic delivery and ferroptosis activation in deep tumor regions. Our comprehensive in vitro and in vivo studies demonstrate the significant therapeutic efficacy of Ec@ZFOY in a 4T1 tumor‐bearing mouse model. The integration of hypoxia‐targeted *E. coli* with magnetic ZFO nanoparticles, loaded with the HIF‐1α inhibitor YC‐1, enables precise navigation and deep tumor penetration under magnetic guidance. The ZFO nanoparticles' POD and GSHox mimicking activities, combined with pH‐responsive YC‐1 release, effectively induce ferroptosis by generating ^•^OH, depleting GSH, and inhibiting HIF‐1α, leading to reduced lipid droplets and enhanced release of PUFAs. Our findings highlight the potential of the Ec@ZFOY biohybrid to overcome the challenges posed by tumor hypoxia and deliver a synergistic approach to tumor therapy through the activation of ferroptosis. This innovative platform offers a promising avenue for enhancing the therapeutic efficacy of cancer treatments and opens new possibilities for the targeted delivery of therapeutics in other disease contexts.

## Author Contributions

**Sijie Shao**: data curation, investigation, methodology, validation, writing – original draft. **Huilan Zhuang**: investigation, methodology, writing – original draft. **Tingjie Bai**: investigation, methodology. **Angelo Homayoun All**: investigation, methodology. **Xuemei Zeng**: conceptualization, data curation, writing – review and editing. **Shuangqian Yan**: conceptualization, data curation, funding acquisition, project administration, supervision, writing – review and editing.

## Conflicts of Interest

The authors declare no conflicts of interest.

## Supporting information




**Supporting File 1**: exp270128‐sup‐0001‐SuppMat.docx

## Data Availability

Data will be made available on request.
