# Supplementary material for: Bacterial Biohybrids With Dual Magnet and Hypoxia Tropism for Ferroptosis Activation in Deep Tumor Regions
Source: Exploration (Beijing). 2026 Feb 10;6(2):20240287. doi: 10.1002/EXP.20240287 (PMC13094518; doi:10.1002/EXP.20240287)
Supplement: Supplementary file 1 — Supporting File 1: exp270128‐sup‐0001‐SuppMat.docx [file EXP2-6-20240287-s001.docx]

**Bacterial biohybrids with dual magnet and hypoxia tropism for ferroptosis activation in deep tumor regions**

*Sijie Shao^1^, Huilan Zhuang^1^, Tingjie Bai^1^, Xuemei Zeng^2^, Jing Luo^3^ Shuangqian Yan^1^**

^1^ Strait Institute of Flexible Electronics (SIFE, Future Technologies), Fujian Key Laboratory of Flexible Electronics, Fujian Normal University and Strait Laboratory of Flexible Electronics (SLoFE), Fuzhou, 350117, China

^2^ Key Laboratory of Microbial Pathogenesis and Interventions of Fujian Province University, Biomedical Research Center of South China, College of Life Sciences, Fujian Normal University, 1 Keji Road, Fuzhou 350117, PR China

^3^ Jing Luo, Department of Chemistry, National University of Singapore, Singapore, 117543, Singapore

* Corresponding authors.

E-mail: [ifeshqyan@fjnu.edu.cn](mailto:ifeshqyan@fjnu.edu.cn)

**KEYWORDS:** Bacterial biohybrids; Ferroptosis; Hypoxia; Tumor penetration

**MATERIALS AND METHODS**

**Materials**. Zinc chloride (≥ 98%), sodium acetate anhydrous, ethylene glycol (EG, > 99%), polyethylene glycol (average M.W. 20000), fluorescein isothiocyanate isomer I (FITC), fluorescein diacetate, 1,3-(5’-hydroxymethyl-2’-furyl)-1-benzylindazole (YC-1), 1,2-diaminobenzene (OPD, AR, 98%), and ethylene imine polymer (PEI, M.W. 1800, 99%) were purchased from Shanghai Aladdin Biochemical Technology Co., Ltd. Iron (III) chloride hexahydrate, iron (II, III) oxide (99.5%, 50 nm), and indocyanine green (ICG) were obtained from Shanghai Macklin Biochemical Technology Co., Ltd. 3,3’,5,5’-tetramethylbenzidine solution (TMB, 1%) and reduced glutathione content assay kit were received from Beijing Solarbio Science Technology Co., Ltd. 6-diamidino-2-phenylindole (DAPI) and calcein AM/PI cell viability/cytotoxicity assay kit were acquired from Shanghai Beyotime Biotechnology Co., Ltd. Hydroxyl radical fluorescent probe BBoxiProbe 026 was purchased from Reibokit. Liperfluo-cell lipid peroxide test kit and Lipi-green were obtained from Dojindo. Cy5 was sourced from Shanghai Yare Biotech, Inc. Annexin V-FITC apoptosis detection kit, Dulbecco’s modified eagle medium (DMEM), and phosphate buffer solution (PBS) were acquired from Shanghai Yeasen Biotechnology Co., Ltd. Fetal bovine serum (FBS) was got from PAN.

**Characterization.** UV-vis-NIR absorption spectra were measured using a UV-8000S spectrophotometer. The morphology of the material was observed on a Hitachi HT7700 transmission electron microscope. Element mappings of Zn_0.16_Fe_1.24_O_4_ were obtained with a high-resolution field emission transmission electron microscope (JEM 2100F). Zeta potential and dynamic light scattering (DLS) were detected by the Malvern Zetasizer Nano ZS (Malvern Instruments, Ltd, Worcestershire, UK). X-ray Diffraction (XRD) was conducted using a Rigaku Miniflex600 X-ray diffractometer. X-ray photoelectron spectroscopy (XPS) spectra were recorded with a Thermo SCIENTIFIC Nexsa a K-Alpha 1063 instrument (Thermo Fisher Scientific, USA).

**Preparation of** **Zn_0.16_Fe_1.24_O_4_ (ZFO) nanoparticles.** Zn_0.16_Fe_1.24_O_4_ nanoparticles were synthesized using a hydrothermal method. Initially, zinc chloride (0.057 g, 0.42 mmol), iron (III) chloride hexahydrate (0.169 g, 0.63 mmol), sodium acetate anhydrous (1.8 g, 21.94 mmol), and polyethylene glycol (0.5 g, 0.025 mmol) were dissolved in ethylene glycol (20 mL) and stirred vigorously for 30 min. The mixture was then transferred into a 100 mL autoclave PTFE-lined autoclave and heated to 200 ℃ for 8 h. After the reaction, the resulting suspension was centrifuged and repeatedly washed with ethanol and water. Finally, the product was dried in a vacuum desiccator at 70 ℃ for 24 h.

**Preparation of ZFOY, Ec@ZFO, and, Ec@ZFOY*.*** To prepare ZFOY, 14 mg of ZFO was dissolved in 10 mL of ultrapure water, followed by the addition of 2 mg of PEI (ZFOP). The mixture was stirred for 6 h. After this reaction, the mixture was centrifuged and repeatedly washed with ultrapure water. Next, 1.4 mg of YC-1 was added to the ZFO solution and stirred for 30 min to obtain the ZFOY aqueous solution. The resulting product was then repeatedly washed with water and vacuum freeze drying for 24 h.

For the preparation of Ec@ZFO and Ec@ZFOY, 2.5 mg of ZFO or ZFOY nanoparticles were dissolved in 900 μL deionized water. Subsequently, 100 μL of an *E. coli* solution (5 × 10^8^ CFU mL^-1^) was added to each nanoparticle solution and co-incubated at 37 ℃ for 2 h with continuous stirring.

**Detection of** ^•^**OH radicals.** The production of ^•^OH was detected using chromogenic reactions of TMB and OPD. Briefly, 50 μL of TMB (1 mg mL^-1^) or OPD (20 mM) was added to 100 μL of ZFO aqueous solution (40 μg mL^-1^) at different pH values (4.5, 6.0, and 7.4) and mixed with 15 µL of 1 mM of H_2_O_2_. After 30 min, the catalytic oxidation was measured using a microplate reader.

**Study on the enzymatic reaction kinetic.** 40 μL ZFO (200 μg mL^-1^) or Fe_3_O_4_ (200 μg mL^-1^) was added into 40 μL pH 6.0 HAc-NaAc buffer solution, then 20 μL of TMB (1 mg mL^-1^) and 100 μL H_2_O_2_ (10，20，40，60, 80 mM) were added into the above solution. UV-vis absorption spectra of oxTMB with a characteristic absorption peak at 652 nm were recorded every 1 min up to 30 min using a microplate reader.

**Evaluation of GSH depletion *in vitro*.** The GSH content was measured by DTNB at the wavelength of 410 nm. The reactions of GSH (200 μg mL^-1^) and ZFO (40 μg mL^-1^) were performed at 37 ℃ for 12 h. After that, DTNB solution (1.5 mg mL^-1^) was added to detect the remaining GSH. Finally, we recorded the absorption of the above-mentioned samples at 412 nm using a microplate reader.

**Characterization of Ec@ZFOY bacterial biohybrids.** Ec@ZFO-Cy5 (200 μL) was co-incubated with fluorescein diacetate (2 μL, 5 mg mL^-1^) for 20 min, then repeatedly washed with deionized water. The solution was then transferred to a confocal petri dish and observed at 488 nm using CLSM. The activity and proliferation of engineered bacteria biohybrids were assessed using fluorescein diacetate staining and flat colony counting. The Ec@ZFOY and *E. coli* were diluted to 1 × 10^3^ CFU mL^-1^, and 20 μL of the bacteria solution was placed on agar plates and incubated at 37 ℃ for 12 h. Additionally, 2 μL of fluorescein diacetate solution was added to each 200 μL (10^8^ CFU mL^-1^) of the bacterial solution, incubated at 37 ℃ for 30 min and observed by CLSM.

**Transwell assay****.** Transwell assays were performed to analyze the migration ability of Ec@ZFOY. A layer of Matrigel was applied to the bottom of the apical chamber of the transwell device. Brieﬂy, standard Matrigel (9 mg mL^-1^) was diluted in serum-free medium at a ﬁnal concentration of 300 μg mL^-1^, and 100 μL of the diluted Matrigel was added to the apical chamber of the 24-well transwell device, followed by gelation at 37 ℃ for 30 min. Then, 700 μL of serum-free medium was added to the bottom chamber, and 200 μL (5 × 10^7^ CFU mL^-1^) of Ec@ZFOY stained with fluorescein diacetate was added to the apical chamber. The device was incubated with or without a magnet or 12 h, and fluorescence was detected.

**pH-responsive release of YC-1.** To investigate the pH-responsive release of YC-1, we dispersed the ZFOY in buffer solutions at varying pH values (4.5, 6.0, and 7.4). The solutions were, incubated at 37 °C with agitation for 6 and 12 h. After incubation, the supernatant was centrifuged, and its UV absorption value was measured.

***In vitro* cytotoxicity assay.** 4T1 cells were cultured in 96-well plates for 24 h at 37 ℃ in a 5% CO_2_ atmosphere and subsequently treated with different formulations: PBS, *E. coli* (1 × 10^6^ CFU mL^-1^), YC-1 (2 μg mL^-1^), ZFO (40 μg mL^-1^), Ec@ZFO (1 × 10^6^ CFU mL^-1^, 40 μg mL^-1^), ZFOY (40 μg mL^-1^), and Ec@ZFOY (1 × 10^6^ CFU mL^-1^, 40 μg mL^-1^) with or without H_2_O_2_ (200 μM). After a 12 h incubation, cell viability was assessed using CCK-8 assay.

**Detection of live/dead cells.** 4T1 cells were cultured in 6-well plates at 37 ℃ with 5% CO_2_ for 24 h and then treated with different formulations (PBS, 1 × 10^6^ CFU mL^-1^ *E. coli*, 2 μg mL^-1^ YC-1, 40 μg mL^-1^ ZFO, 1 × 10^6^ CFU mL^-1^ Ec@ZFO, 40 μg mL^-1^ ZFOY, 1 × 10^6^ CFU mL^-1^ Ec@ZFO) along with H_2_O_2_ (200 μM). After co-incubation at 37 °C for 12 h, the cells were stained with Calcein AM and PI for 30 min. Subsequently, the 4T1 cells were washed with PBS and observed under a fluorescent microscope.

***In vitro* cellular uptake assay.** 4T1 cells were cultured in six-well plates for 24 h at 37 ℃ in a 5% CO_2_ atmosphere. The medium was replaced with fresh medium containing FITC-labelled ZFO NPs (40 μg mL^-1^) and incubated for 0, 2, 4, 8, 12, and 24 h. The cells were then washed twice with PBS and analyzed using flow cytometry.

**Detection of intracellular lipid droplets.** 4T1 cells were cultured in a confocal dish for 24 h and subjected to the following treatments: (1) PBS; (2) *E. coli* (1 × 10^6^ CFU mL^-1^); (3) YC-1 (2 μg mL^-1^); (4) Ec@ZFO (1 × 10^6^ CFU mL^-1^); and (5) Ec@ZFOY (1 × 10^6^ CFU mL^-1^). The cells were then stained with Lipi-Green and examined using CLSM.

**Detection of LPO.** 4T1 cells were seeded into confocal dish and cultured for 24 h. Fresh medium (control) or medium containing *E. coli* (1 × 10^6^ CFU mL^-1^), YC-1 (2 μg mL^-1^), ZFO (40 μg mL^-1^), Ec@ZFO (1 × 10^6^ CFU mL^-1^, 40 μg mL^-1^), ZFOY (40 μg mL^-1^) or Ec@ZFOY (1 × 10^6^ CFU mL^-1^, 40 μg mL^-1^) was added and co-incubated for 12 h. Afterward, the cells were washed with serum-free medium and stained with Liperfluo (200 μM) at 37 °C for 30 min. Finally, the stained cells were observed by using CLSM. (Liperfluo: excitation wavelength = 488 nm, emission wavelength = 500-550 nm)

**Cell apoptosis analysis.** 4T1 cells were cultured in 6-well plates at 37 ℃ with 5% CO_2_ for 24 h and then treated with different treatments (PBS, 1 × 10^6^ CFU mL^-1^ *E. coli*, 2 μg mL^-1^ YC-1, 40 μg mL^-1^ ZFO, 1 × 10^6^ CFU mL^-1^ Ec@ZFO, 40 μg mL^-1^ ZFOY, 1 × 10^6^ CFU mL^-1^ Ec@ZFO, and 1 × 10^6^ CFU mL^-1^ Ec@ZFOY) with or without H_2_O_2_ (200 μM). After co-incubated for 12 h, 4T1 cells were stained with the apoptosis detection kit, followed by flow cytometric analysis.

**Penetration of bacterial biohybrids in 3D 4T1 spheroids.** 4T1 cells were digested and seeded into ultra-low attachment 96-well plates, followed by a 4-day incubation to form 3D spheroids. These spheroids were divided into four groups and incubated for 6 h with different formulations: ZFO/FITC, Ec@ZFO/FITC, ZFO/FITC + magnet, and Ec@ZFOY + magnet. The fluorescence of the 3D spheroids was then imaged using CLSM.

***In vivo* biodistribution.** All animal experiments were implemented in accordance with protocols approved by the Animal Experimental Ethics Committee of Fujian Normal University. To establish the 4T1 tumor model, 100 μL of 4T1 cells (10^6^ cells) were injected subcutaneously into the right thigh of mice. Once the tumor volume reached approximately 200 mm^3^**,** the tumor-bearing Balb/c mice were randomly divided into four groups and administrated intravenous injections (n = 3) as follows: (1) ZFO@ICG (2 mg mL^-1^, 100 μL); (2) ZFO@ICG (2 mg mL^-1^, 100 μL) + magnet; (3)Ec@ZFO/ICG (5 × 10^7^ CFU mL^-1^); and (4) Ec@ZFO/ICG (5 × 10^7^ CFU mL^-1^) + magnet. Whole-body fluorescence images were captured at various time points using an *in vivo* imaging system (IVIS, RWD MOIS HT). After 24 h, the mice were euthanized, and their tumors and major organs were harvested for *ex vivo* imaging.

***In vivo* tumor therapy.** To establish the 4T1 tumor model, 100 μL of 4T1 cells (1 × 10^6^ cells) were injected into the subcutaneous tissue of the right thigh of mice. Once the tumor volume reached approximately 200 mm^3^**,** tumor-bearing BALB/c mice were randomly divided into eight groups and intravenously injected (n = 5): (1) PBS (control); (2) *E. coli* (5 × 10^7^ CFU mL^-1^, 100 μL); (3) ZFO (2 mg mL^-1^, 100 μL) + magnet; (4) ZFOY (2 mg mL^-1^, 100 μL); (5) ZFOY (2 mg mL^-1^, 100 μL) + magnet; (6) Ec@ZFO (5 × 10^7^ CFU mL^-1^, 2 mg mL^-1^, 100 μL) + magnet; (7) Ec@ZFOY (5 × 10^7^ CFU mL^-1^, 2 mg mL^-1^); and (8) Ec@ZFOY (5 × 10^7^ CFU mL^-1^, 2 mg mL^-1^) + magnet. The magnet was removed after two days. Tumor size and body weight were measured every two days, and tumors were photographed and weighed when mice were euthanized following 14 days of treatments. The excised tumors were sliced and subjected to histological and immunohistochemical analysis.

***In vivo* toxicity evaluation.** For the hemolysis test, blood was collected from the eyeball, washed with PBS, and centrifuged to obtain red blood cells, which were then suspended in PBS. The red blood cell suspensions were treated with 0.5% Triton X-100 (as the positive control), PBS, and various concentrations of ZFO, incubated at 37 ℃ for 1 h, centrifuged, and photographed. Subsequently, the supernatant was transferred to a 96-well plate, and the absorbance at 577 nm was measured.

For the long-term toxicity test, ten healthy Balb/c mice were divided into two groups (n = 5): PBS and Ec@ZFOY (5 × 10^7^ CFU mL^-1^, 2 mg mL^-1^, 100 μL). The mice were weighed every five days. After 60 days, blood was collected for a routine examination, and the major organs (heart, liver, spleen, lung, and kidney) were sectioned for histological analysis.

**Animal model.** Balb/c mice (female, 4-6 weeks) were purchased from Hua Fu Kang Biotechnology Co., Ltd. (Beijing, China) and raised in SPF animal rooms. All animal procedures were implemented by protocols approved by the Animal Experimental Ethics Committee of Fujian Normal University for animal experimentation and care (Approval No. IACUC-20220004).

**Statistical analysis.** Quantitative data were presented as mean ± standard error of the mean (SEM). All experiments were repeated three times or more. Statistics were analyzed by Student’s t-test (two-tailed) and one-way ANOVA, followed by Tukey's multiple comparisons test using GraphPad Prism 8.0. Statistical significance was denoted by an asterisk. P values of 0.05 or less were considered statistically significant (ns: not significant, *P < 0.05, **P < 0.01, ***P < 0.001, and ****P < 0.0001).


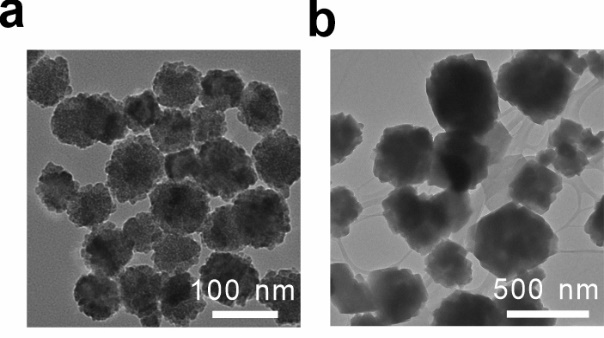


**Figure S1**. (a) TEM image of ZFO-2. (b) TEM image of ZFO-3. Scale bars are 100 nm and 500 nm for ZFO-2 and ZFO-3, respectively.


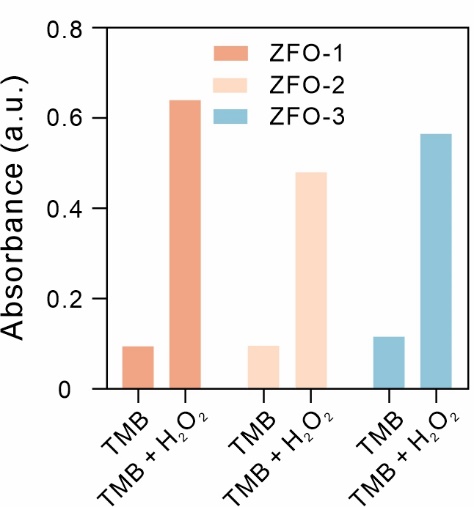


**Figure S2**. Catalytic ability of different zinc-doped materials.


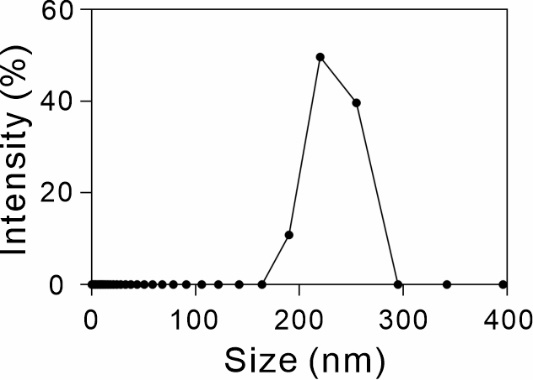


**Figure S3**. Hydrodynamic diameter of ZFO.

**
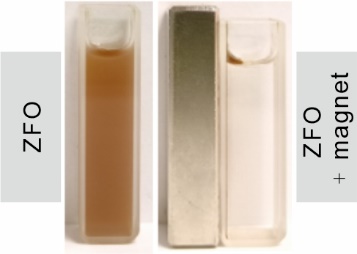
**

**Figure S4**. Pictures of ZFO attracted by magnets.


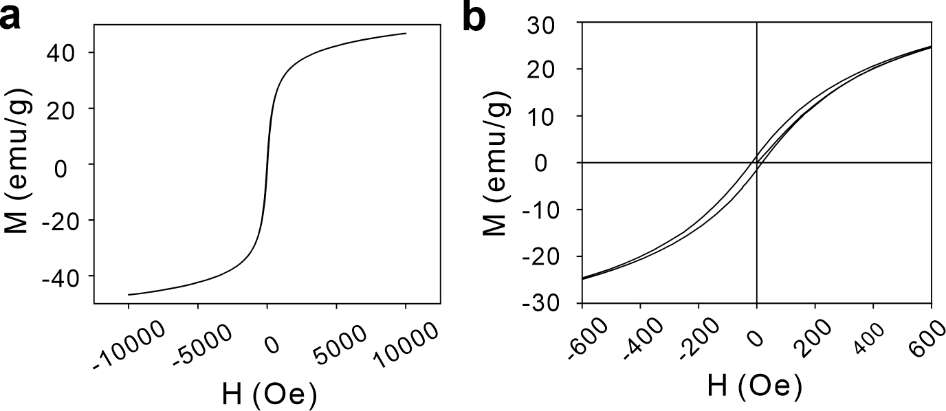


**Figure S5**. (a, b) Magnetic performance of Fe_3_O_4_ measured by VSM at room temperature.

**
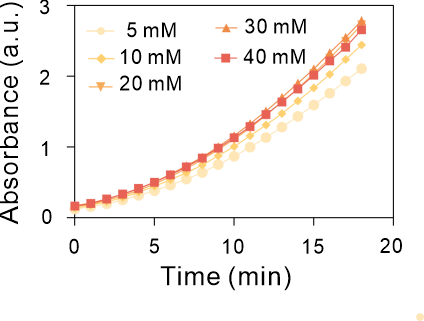
**

**Figure S6**. Time-course TMB absorbance (652 nm) of ZFO solution after the addition of varying concentrations of H_2_O_2_.


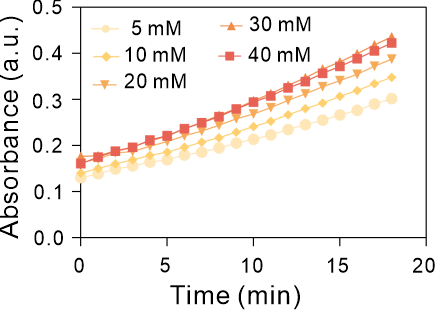


**Figure S7**. Time-course TMB absorbance (652 nm) of Fe_3_O_4_ solution after the addition of various concentrations of H_2_O_2_.


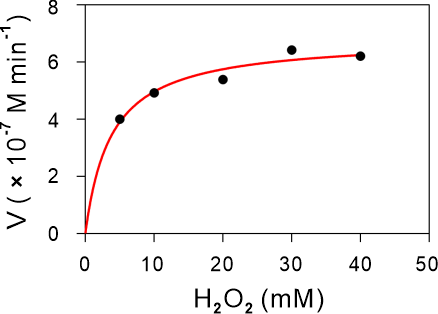


**Figure S8**. Michaelis-Menten kinetic analysis for Fe_3_O_4_ with H_2_O_2_ as a substrate.


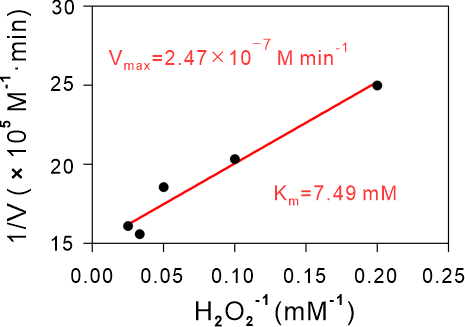


**Figure** **S9**. Lineweaver-Burk plot for Fe_3_O_4_ with H_2_O_2_ as a substrate.

_
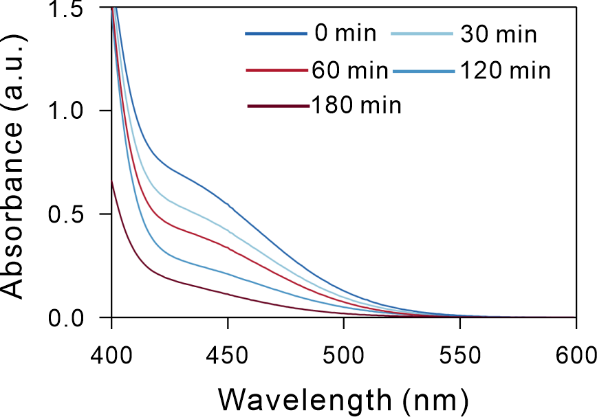
_

**Figure** **S10**. Time-dependent GSH depletion by ZFO.


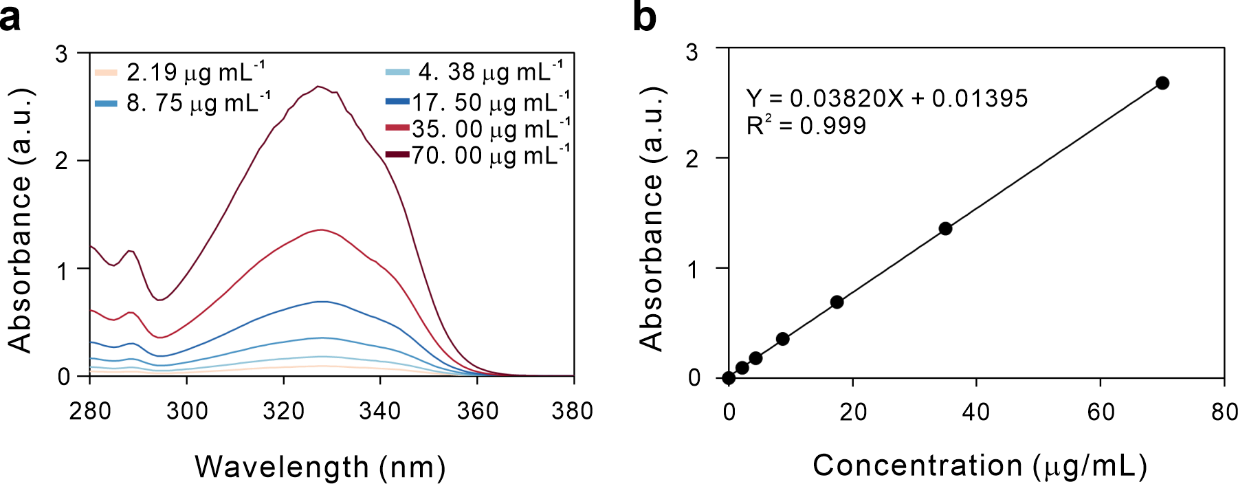


**Figure S11**. (a) UV-Vis absorption curves of YC-1 with different concentrations. (b) The standard curve of YC-1.


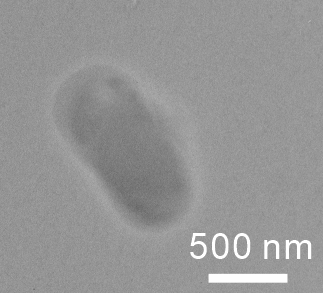


**Figure S12**. TEM image of *E. coli*.


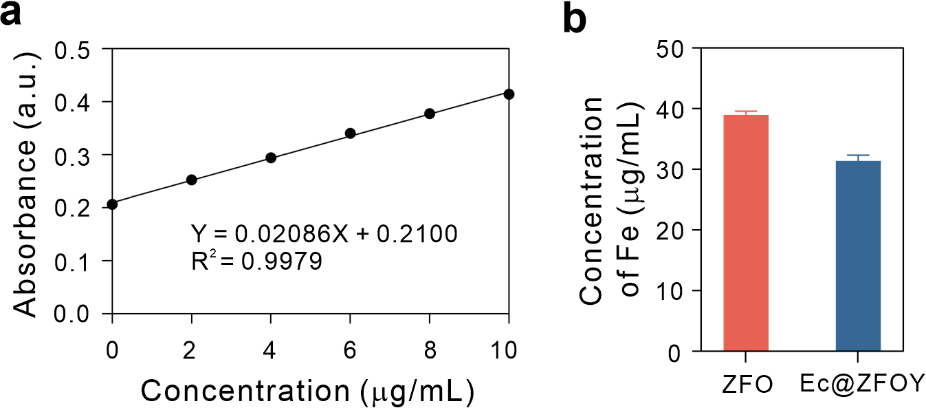


**Figure S13**. (a) The standard curve of Fe concentration. (b) Iron content in various substances.


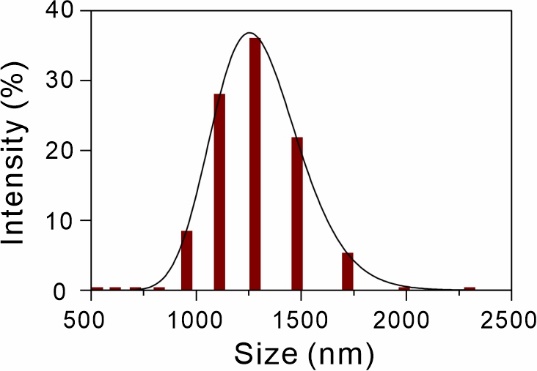


**Figure S14**. Hydrodynamic diameter of *E. coli*.


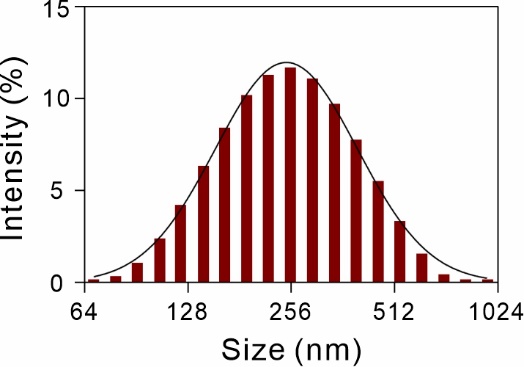


**Figure S15**. Hydrodynamic diameter of ZFOY.


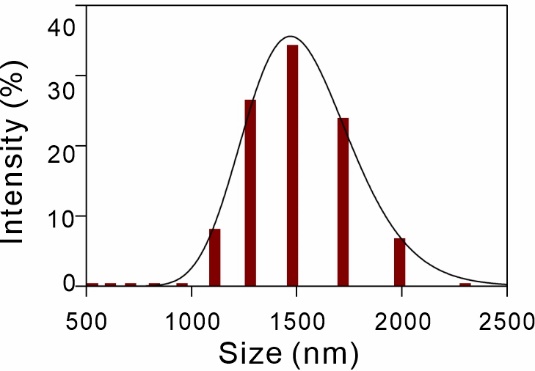


**Figure S16**. Hydrodynamic diameter of Ec@ZFOY.


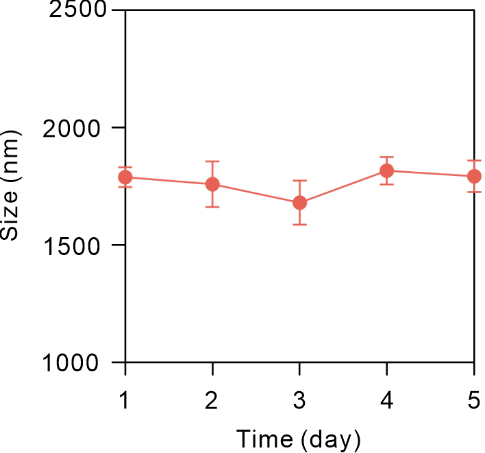


**Figure S17**. Long-term hydrodynamic diameter of Ec@ZFOY in DMEM contained FBS. (n = 3)


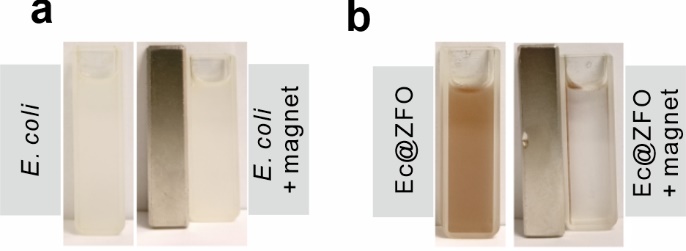


**Figure S18**. Pictures of bacterial biohybrids controlled by a magnet. (a) *E. coli*. (b) Ec@ZFO.

| The group | *E. coli* + Magnet | Ec@ZFOY | Ec@ZFOY + Magnet |
| --- | --- | --- | --- |
| The valid displacement (μm) | 8.05838 | 28.79963 | 121.9493 |

**Table S1**. The valid displacement of different groups.


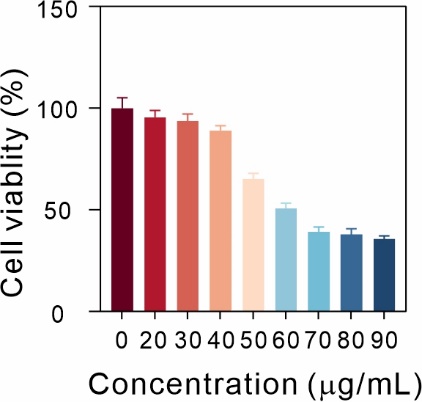


**Figure S19**. CCK-8 assay of 4T1 cells after treatment with different concentrations of ZFO.


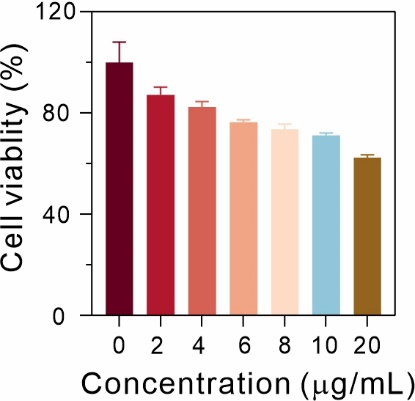


**Figure S20**. CCK-8 assay of 4T1 cells after treatment with different concentrations of YC-1.


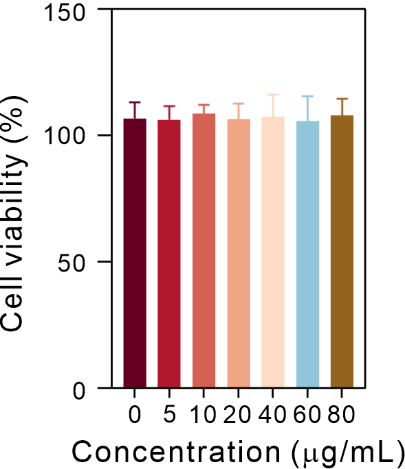


**Figure S21**. Cell viability of NIH/3T3 cells treated with different concentrations of ZFO (n = 6).


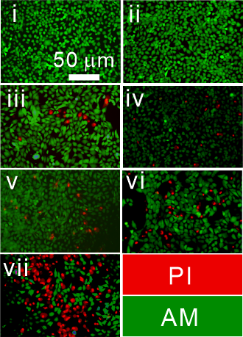


**Figure S22**. CLSM images of 4T1 cells stained with CalceinAM (green)/PI (red) after various treatments. (i) PBS, (ii) *E. coli*, (iii) YC-1, (iv) ZFO, (v) Ec@ZFO, (vi) ZFOY, (vii) Ec@ZFOY.


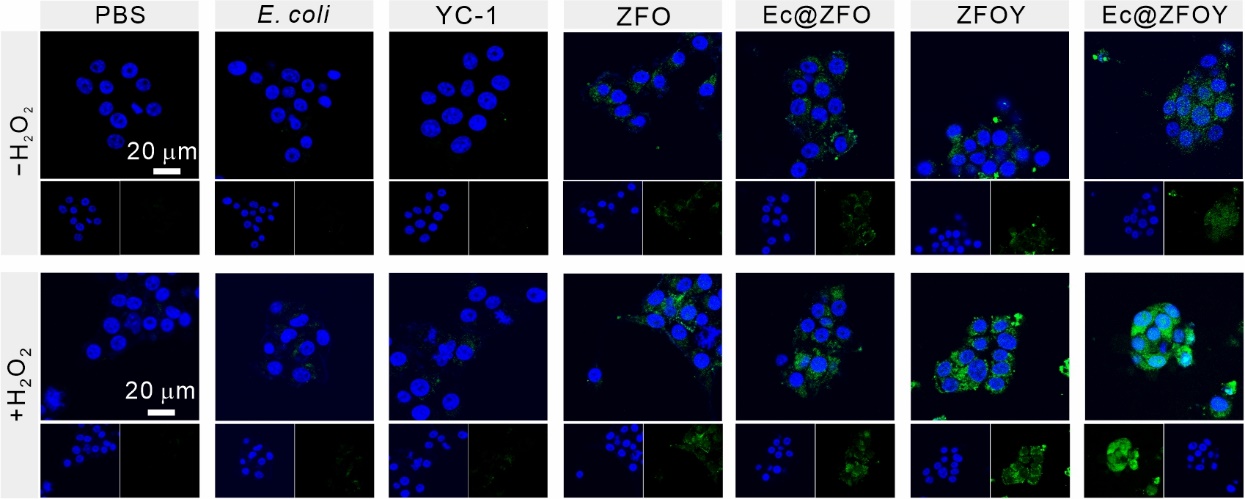


**Figure S23**. Confocal microscopy images of cells with various treatments of intracellular ^•^OH stained with BBoxiProbe O26.


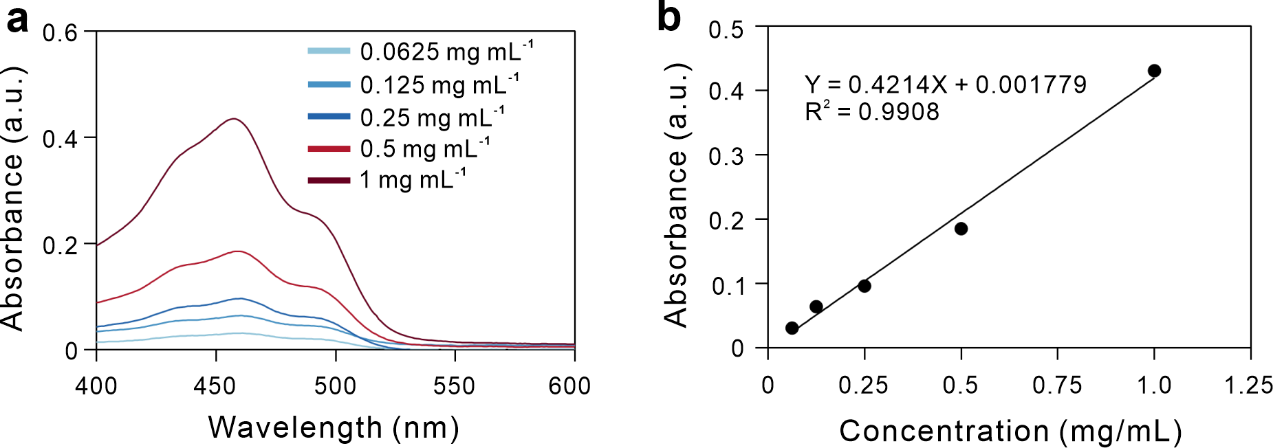


**Figure S24**. (a) UV-Vis absorption curves of FITC with different concentrations. (b) The standard curve of FITC.


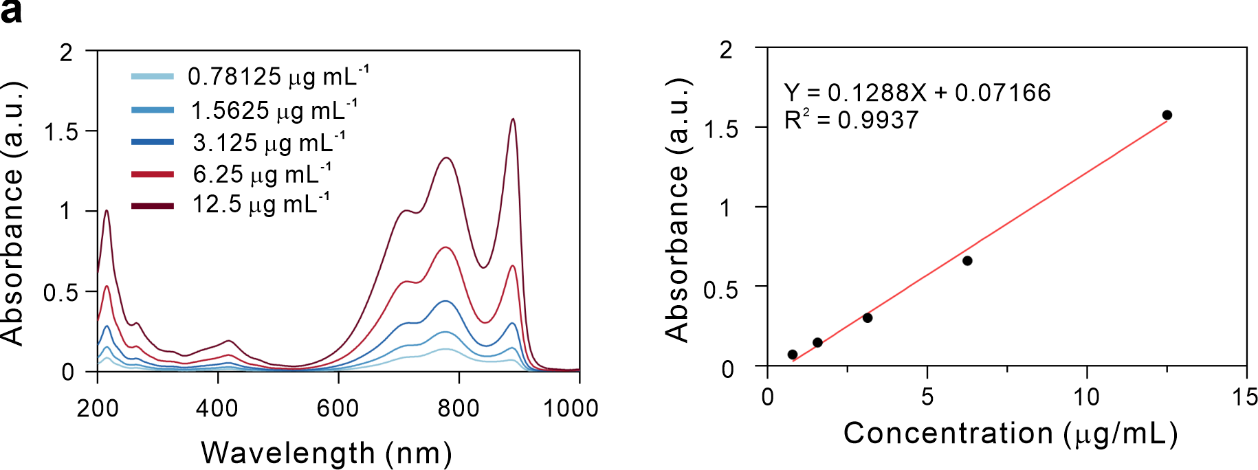


**Figure S25**. (a) UV-Vis absorption curves of ICG with different concentrations. (b) The standard curve of ICG.


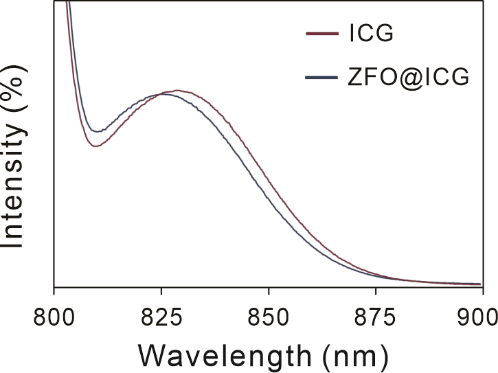


**Figure S26**. The fluorescence spectra of ICG and ZFO/ICG.


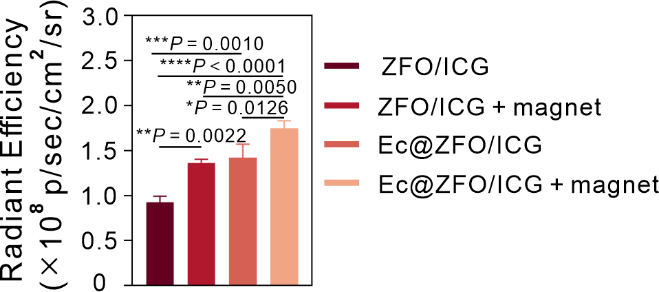


**Figure S27.** A separate figure of the radiant efficiency of tumor tissues from four groups.


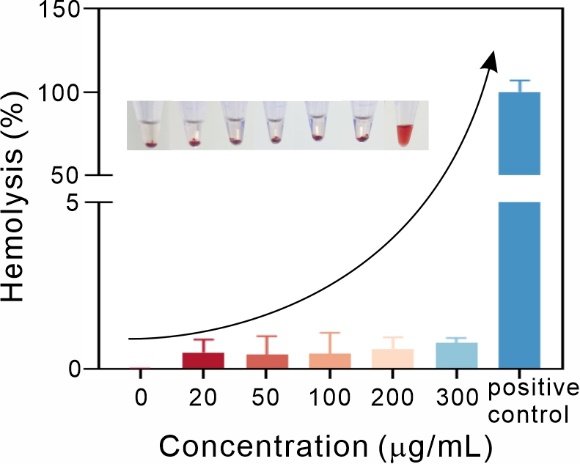


**Figure S28**. Hemolytic experiment of ZFO with different concentration.
